# Overexpression of a type III PKS gene affording novel violapyrones with enhanced anti-influenza A virus activity

**DOI:** 10.1186/s12934-018-0908-9

**Published:** 2018-04-12

**Authors:** Lukuan Hou, Huiming Huang, Huayue Li, Shuyao Wang, Jianhua Ju, Wenli Li

**Affiliations:** 1 Key Laboratory of Marine Drugs, Ministry of Education of China, School of Medicine and Pharmacy, Ocean University of China, Qingdao, 266003 China; 2 Laboratory for Marine Drugs and Bioproducts of Qingdao National Laboratory for Marine Science and Technology, Qingdao, 266237 China; 3 CAS Key Laboratory of Marine Bio-resources Sustainable Utilization, Guangdong Key Laboratory of Marine Materia Medica, RNAM Center for Marine Microbiology, South China, Sea Institute of Oceanology, Chinese Academy of Sciences, 164 West Xingang Road, Guangzhou, 510301 China

**Keywords:** Violapyrones (VLPs), Overexpression, Type III polyketide synthase (PKS), Anti-influenza A (H1N1) virus activity

## Abstract

**Background:**

Type III polyketide synthases (PKSs) are simple homodimer ketosynthases that distribute across plants, fungi, and bacteria, catalyzing formation of pyrone- and resorcinol-types aromatic polyketides with various bioactivities. The broad substrate promiscuity displayed by type III PKSs makes them wonderful candidates for expanding chemical diversity of polyketides.

**Results:**

Violapyrone B (VLP B, **10**), an α-pyrone compound produced by deepsea-derived *Streptomyces somaliensis* SCSIO ZH66, is encoded by a type III PKS VioA. We overexpressed VioA in three different hosts, including *Streptomyces coelicolor* M1146, *Streptomyces sanyensis* FMA as well as the native producer *S. somaliensis* SCSIO ZH66, leading to accumulation of different violapyrone compounds. Among them, *S. coelicolor* M1146 served as the host producing the most abundant violapyrones, from which five new (**2**–**4**, **7** and **12**) and nine known (**1**, **5**, **6**, **8**–**11**, **13** and **14**) compounds were identified. Anti-influenza A (H1N1) virus activity of these compounds was then evaluated using ribavirin as a positive control (IC_50_ = 112.9 μM), revealing that compounds **11**–**14** showed considerable activity with IC_50_ values of 112.7, 26.9, 106.7 and 28.8 μM, respectively, which are significantly improved as compared to that of VLP B (**10**) (IC_50_ > 200 μM). The productions of **10** and **13** were increased by adding P450 inhibitor metyrapone. In addition, site-directed mutagenesis experiment led to demonstration of the residue S242 to be essential for the activity of VioA.

**Conclusions:**

Biological background of the expression hosts is an important factor impacting on the encoding products of type III PKSs. By using *S. coelicolor* M1146 as cell factory, we were able to generate fourteen VLPs compounds. Anti-H1N1 activity assay suggested that the lipophilic nature of the alkyl chains of VLPs plays an important role for the activity, providing valuable guidance for further structural optimization of VLPs.

**Electronic supplementary material:**

The online version of this article (10.1186/s12934-018-0908-9) contains supplementary material, which is available to authorized users.

## Background

Type III polyketide synthases (PKSs) catalyze carbon–carbon bond formation through a complete series of decarboxylation, condensation, and cyclization reactions with a single active site [1]. The diversity of type III PKS-catalyzed reactions is ascribed to selectivity of starter and extender units, number of condensations, and intramolecular cyclization manners [2]. Notably, many type III PKSs display broad substrate promiscuity, and can recognize unnatural substrates to generate novel unnatural products, thus making them fantastic candidates for enzymatic engineering to expand chemical diversity of polyketides [3–8].

Crystal structural studies have shown that type III PKSs share a common three-dimensional structure and catalytic machinery which contains a conserved Cys-His-Asn catalytic triad [9, 10]. Substitution of non-catalytic residues located at the substrate binding, CoA binding and cyclization pockets can change the preference of substrates, number of condensation as well as cyclization manner and thus have impacts on their product selectivity [11]. A lot of mutagenesis studies with the aim of broadening substrate specificity have been carried out in plant-derived type III PKSs. For example, the S338V variant of chalcone synthase (CHS) from *Scutellaria baicalensis* produced octaketides SEK4/SEK4b from eight molecules of malonyl-CoA instead of condensing 4-coumaroyl-CoA with three molecules of malonyl-CoA to generate naringenin chalcone as did the wild type CHS [12]. Substitution of N222 with Gly in octaketide synthase (OKS) from *Aloe arborescens* led to accumulation of a novel C_20_ decaketide SEK15 in addition to the C21 heptaketide chalcone that is produced by the wild type OKS [13]. The L214I variant of *Vitis vinifera* stilbene synthase (VvSTS) bears an increased substrate binding pocket and a decreased cyclization pocket compared with those in the wild type enzyme, resulting in production of short-chain polyketides with improved efficiency but absence of long-chain polyketides; conversely, the sizes of both pockets in the T197A variant were increased, thus leading to generation of five new polyketides which are not produced by the wild type VvSTS [14].

In contrast, only a handful of mutagenesis studies have been reported for prokaryotic type III PKSs, among which the active-site cavity-forming residue Y224 in Sg-RppA from *Streptomyces griseus* [15] and Sc-RppA from *S. coelicolor* [16] has been studied the most. In both enzymes, Y224 was demonstrated to be important for starter substrate selection, but Sc-RppA showed a higher tolerance towards certain amino acid changes of Y224 than Sg-RppA. Mutants of Y224 were thus generated, which preferentially recognize unnatural acyl-CoA such as acetyl-CoA, acetoacetyl-CoA, hexanoyl-CoA and benzoyl-CoA instead of malonyl-CoA as starter substrate [15, 16]. Another example is the mutagenesis of Gcs from *S. coelicolor*, which disclosed H261 and M274 are critical in controlling the substrate specificity and/or catalytic efficiency, as the H261A and M274A variants were capable of producing significantly increased amount of triketide pyrones in comparison to the wild-type Gcs [17].

**Fig. 1:**
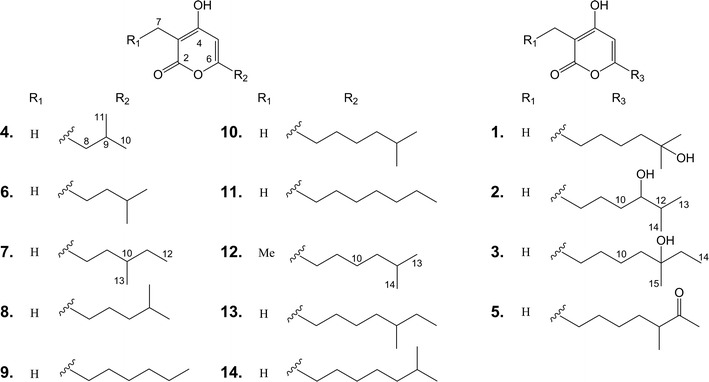
Structures of violapyrones (**1**–**14**). **1**, VLP F; **5**, VLP D; **6**, VLP J1; **8**, VLP A; **9**, VLP J; **10**, VLP B; **11**, VLP I; **13**, VLP C; **14**, VLP H. **2**, **3**, **4**, **7** and **12** are novel VLP analogues

Violapyrones (VLPs) are a group of α-pyrone compounds with antibacterial and anticancer activities [18–20]. Previously, we activated the VLP biosynthetic gene cluster via deletion of the global regulatory gene *wblA*_*so*_ in deepsea-derived *Streptomyces somaliensis* SCSIO ZH66, leading to isolation of VLP B (**10**) (Fig. 1) [18]. This cluster is composed of a type III PKS gene *vioA* and a negative regulatory gene *vioB*. By inactivation of *vioB*, another four VLP compounds (VLP A, J, C and H) were obtained, which were subjected to evaluation of anti-MRSA (methicillin-resistant *Staphylococcus aureus*, MRSA) activity, demonstrating that the length of the alkyl side chains of VLPs played an essential role for the anti-MRSA activity [18].

The broad substrate promiscuity displayed by VioA inspired us to explore its synthetic potentials. Herein, we describe generation of VLP compounds (**1**–**14**) by over-expression of VioA in three different hosts, among which five (**2**–**4**, **7** and **12**) are new. The following evaluation of anti-influenza A (H1N1) virus activity indicated that four (**11**–**14**) exhibited improved anti-H1N1 activity compared to that of VLP B (**10**).

## Results

### Phylogenetic analysis of VioA and distribution of *vio* cluster

**Fig. 2:**
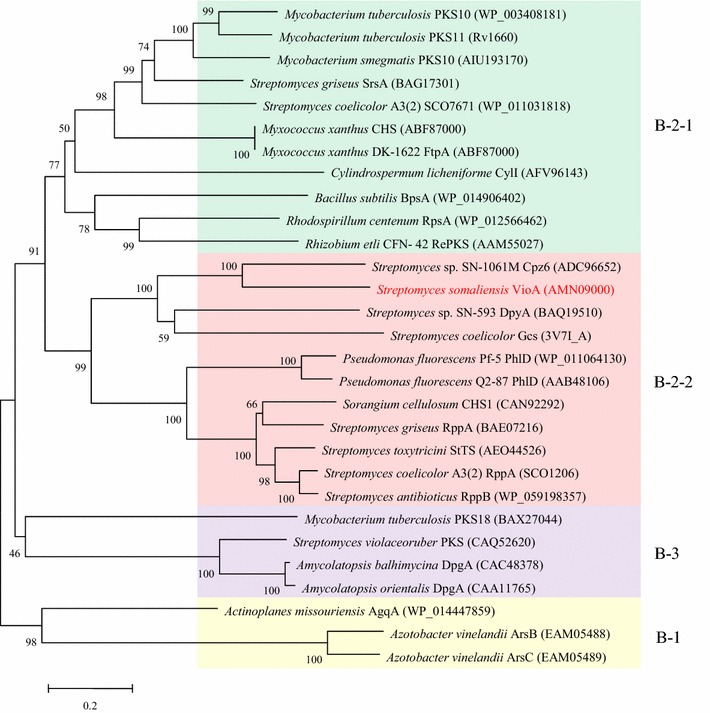
Phylogenetic analysis of VioA with characterized bacterial type III PKSs. Sequences were aligned with ClustalW, and the tree was constructed by using the neighbor joining method. The reliability of the tree was measured by bootstrap analysis with 1000 replicates. Scale: number of substitutions per nucleotide. Colored backgrounds indicate enzyme groups: yellow, B-1 PKS; green, B-2-1 PKS; pink, B-2-2 PKS; purple, B-3 PKS [2]

To better understand the function of VioA, phylogenetic analysis was performed to with other characterized bacterial type III PKSs. As shown in Fig. 2, VioA belongs to the B2-2 clade [2], which preferentially uses short- and medium-chain (C_2_–C_12_) acyl-CoA as starter. VioA is closest to Cpz6 from the caprazamycin biosynthetic gene cluster [21], and they are clustered with DpyA and Gcs, which are proposed to recognize both CoA- and ACP-tethered β-keto acids from branched-chain or straight-chain fatty acid metabolism as starters, and to generate pyrones by lactonization of a linear polyketide intermediate [2]. In contrast, the members of the other subclade in B2-2 use malonyl CoA as both starter and extender unit to give scaffolds of pyrones and resorcinols by lactonization or Claisen-, aldol-type cyclization, respectively. We further mined the *vioAB* locus from other *Streptomyces* genomes in Genbank, and found another 21 *Streptomyces* genomes harboring *vioAB* homologous *loci* (Additional file 1: Table S3). Notably, most of them are located in a linear plasmid, probably contributing to their horizontal gene transfer during evolution.

### Overexpression of *vioA* in different *Streptomyces* strains

**Fig. 3:**
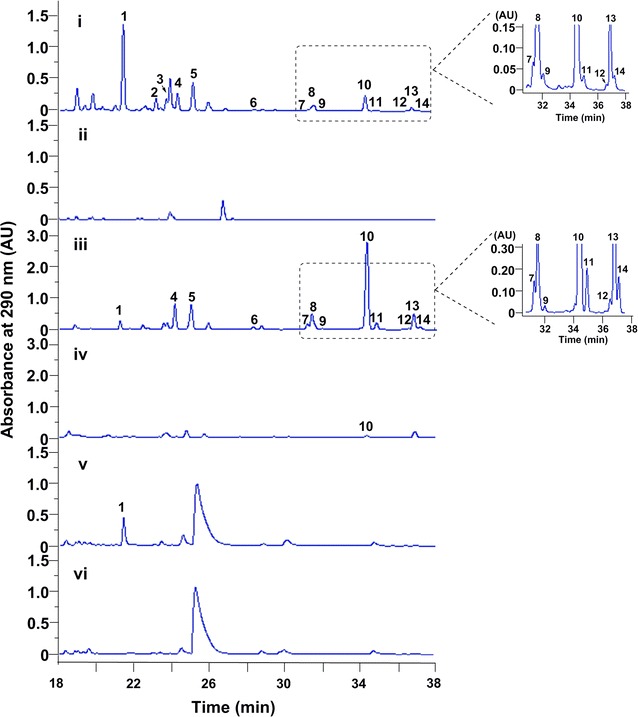
HPLC traces of the fermentation broths from overexpression strains. (i) *S. coelicolor* M1146/pWLI807; (ii) *S. coelicolor* M1146/pWLI806; (iii) *S. somaliensis* SCSIO ZH66/pWLI807; (iv) *S. somaliensis* SCSIO ZH66/pWL806; (v) *S. sanyensis* FMA/pWLI807; (vi) *S. sanyensis* FMA/pWLI806

Type III PKSs capture acyl-CoA substrates from primary metabolism. Considering the variety of the acyl-CoA pools in different biological backgrounds, the VLP gene cluster was overexpressed in three different hosts, including the general heterologous expression host *S. coelicolor* M1146 [22], the marine-derived *Streptomyces sanyensis* FMA [23] as well as the native producer *S. somaliensis* SCSIO ZH66 [18, 24]. To get rid of the negative regulatory function of *vioB* [18], the *vioA* gene was put under the control of the constitutive promoter P_gapDH_ followed by introduction into different hosts as described in the materials and methods section. HPLC analysis of the fermentation broths showed that in addition to VLP B (**10**), several other VLPs compounds were also accumulated in M1146/pWLI807 (Fig. 3i) and ZH66/pWLI807 (Fig. 3iii), conversely, only compound **1** was accumulated in FMA/pWLI807 (Fig. 3v), indicating the significant influence of the expression hosts on the products. Their relative yields in each host were indicated in Additional file 1: Figure S1.

### Identification of the accumulated VLPs in the overexpression strains

From the large scale fermentations of the overexpression strain M1146/pWLI807, compounds **1**–**14** were isolated and identified via detailed NMR spectroscopic analysis.

**Fig. 4:**
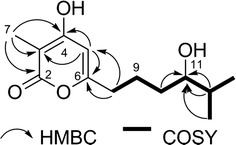
Key HMBC and COSY correlations of **2** in DMSO-*d*_*6*_

Compound **2** was obtained as a colorless oil, and the molecular formula C_13_H_20_O_4_ was deduced from the [M+H]^+^ molecular ion peak at *m/z* 241.1432 in the HR-ESIMS (calcd for 241.1362). The structure of **2** was determined by 1D (^1^H and ^13^C) and 2D (COSY, HSQC and HMBC) NMR data. The ^13^C and HSQC spectra displayed three oxygenated quaternary carbons (δ_C_ 162.1–166.9), an olefinic methine carbon (δ_C_ 100.2), a quaternary carbon (δ_C_ 95.5), a hydroxylatedmethine carbon (δ_C_ 74.0), three methylene carbons (δ_C_ 32.7–33.2), a methine carbon (δ_C_ 23.2) and three methyl carbons (δ_C_ 8.3–18.9). Analysis of the COSY spectrum of **2** suggested a proton spin system from H-8 (δ_H_ 2.37) to H-13 (δ_H_ 0.81), constructing an aliphatic chain (Fig. 4). The location of the methyl group (δ_H_ 1.72) at C-3 (δ_C_ 95.5) was readily determined by its HMBC correlations (Fig. 4) with two oxygenated quaternary carbons C-2 (δ_C_ 165.4) and C-4 (δ_C_ 166.9), and as well as with C-3. Similarly, the olefinic methine proton H-5 (δ_H_ 5.93) showed HMBC correlations with C-3, C-4, C-6 (δ_C_ 162.1) and C-8 (δ_C_ 32.7) (Tables 1 and 2). From these HMBC correlations together with the fact that **2** needs to form a ring to satisfy the unsaturation number, an α-pyrone ring was constructed. In addition, the HMBC correlation from H-5 to C-8 confirmed the connectivity of the α-pyrone ring to the aliphatic chain. Thus, the structure of **2** was determined as 3-methyl-4-hydroxy-6-(11-hydroxy-12-methylhexyl)-2*H*-pyran-2-one, named as VLP L. The yield of **2** was 3.14 mg/L.

**Table 1:** ^1^H NMR (500 MHz) data for compounds **2**–**4**, **7** and **12** in DMSO-*d*_*6*_ (*J* in Hz)

Position	**2**	**3**	**4**	**7**	**12**
2					
3					
4					
5	5.93, s, 1H	5.94, s, 1H	5.86, s, 1H	5.89, s, 1H	5.88, s, 1H
6					
7	1.72, s, 3H	1.72, s, 3H	1.69, s, 3H	1.70, s, 3H	2.24, dd (14.5, 7.0), 2H
8	2.37, m, 2H	2.38, t (7.5), 2H	2.21, d (6.5), 2H	2.34, m, 2H	2.35, t (7.0), 2H
9	1.35, m, 2H	1.49, m, 2H	1.89, m, 1H	1.52, m, 2H	1.49, m, 2H
10	1.27, m, 2H	1.30, m, 2H	0.88, d (6.5), 3H	1.32, m, 1H	1.26, m, 2H
11	3.15, m, 1H	1.30, m, 2H	0.88, d (6.5), 3H	1.13, m, 2H	1.17, m, 2H
12	1.50, m, 1H			0.83, m, 3H	1.50, m, 1H
13	0.81, dd (10.0, 4.0), 3H	1.32, m, 2H		0.84, m, 3H	0.84, d (6.5), 3H
14	0.81, dd (10.0, 4.0), 3H	0.78, t (7.5), 3H			0.84, d (6.5), 3H
15		0.98, m, 3H			
16					0.92, t (6.5), 3H

**Table 2:** ^13^C NMR (125 MHz) data for compounds **2**–**4**, **7** and **12** in DMSO-*d*_*6*_

Position	**2**	**3**	**4**	**7**	**12**
2	165.4	166.0	165.4	165.4	164.7
3	95.5	96.4	94.7	100.9	101.4
4	166.9	166.5	168.0	168.2	166.8
5	100.2	100.4	101.9	95.0	100.2
6	162.1	162.6	160.3	161.6	161.6
7	8.3	9.1	8.4	8.7	15.9
8	32.7	33.3	41.6	30.1	32.4
9	32.8	27.6	26.0	33.0	26.5
10	33.2	23.3	21.7	28.4	25.8
11	74.0	41.3	21.7	28.3	37.7
12	23.2	71.1		10.8	27.0
13	18.9	34.5		18.5	22.2
14	17.6	8.9			22.2
15		26.8			
16					12.5

Structure elucidation of compounds **3**, **4**, **7** and **12** was straightforward because of their close structural relationships with **2**. Thus, by combination of NMR assignment with corresponding HR-ESIMS data, compounds **3**, **4**, **7** and **12** were identified as new VLP derivatives, named VLPs M-P, respectively. The ^1^H and^13^C chemical shift values of five new compounds (**2**–**4**, **7** and **12**) are shown in Tables 1 and 2, respectively. The key HMBC and COSY correlations of **3**, **4**, **7** and **12** were described in supporting information (Additional file 1: Figures S4, S5, S8, S13). The yields of **3**, **4**, **7** and **12** were 1.75, 6.63, 0.29 and 0.13 mg/L, respectively.

Compounds **1**, **5**, **6**, **8**–**11**, **13** and **14** were identified as VLPs F, D, J1, A, J, B, I, C and H respectively, by comparison of ^1^H data with those reported in the literatures [18–20, 25] (Additional file 1: Figures S2, S6, S7, S9, S10, S11, S12, S14, S15), among which **8**–**10** and **13**–**14** had been identified from *S. somaliensis* SCSIO ZH66 mutant strains before [18]. Their yields of compounds **1**, **5**, **6**, **8**–**11**, **13** and **14** were 25.40, 7.50, 0.75, 4.73, 0.29, 8.53, 0.46, 2.97 and 0.25 mg/L, respectively.

### Anti-H1N1 activity of VLPs

Before evaluating the anti-H1N1 activity of compounds **1**–**14**, the cytotoxicity of compounds **1**–**14** in MDCK cell was evaluated by MTT assay [26]. The results in Table 3 showed that compounds **1**–**14** exhibited no significant cytotoxicity and CC_50_ value for compounds **1**–**14** were more than 1400 μM. Compounds **1**–**14** were evaluated for their anti-H1N1 activity by using CPE inhibition assay [27]. As shown in Table 3, compounds **11** and **13** showed moderate anti-H1N1 activities with IC_50_ values of 112.7 and 106.7 μM, respectively, which is comparable to that of the positive control ribavirin (IC_50_ = 112.9 μM); delightedly, compounds **12** and **14** exhibited stronger anti-H1N1 activities than ribavirin up to fourfold, with the IC_50_ values of 26.9 and 28.8 μM, respectively. In contrast, compounds **1**–**10** were inactive against H1N1 virus up to the concentration of 200 μM. Comparing the structures and bioactivities of these compounds, we proposed that the length and polarity of the alkyl side chains at C-3 and C-6 play essential roles for the antiviral activity, in which the one containing longer alkyl side chain with lower polarity gives better activity.

**Table 3:** Inhibition effects of compounds **1**–**14** on H1N1 virus multiplication in vitro

Compounds	IC_50_ (μM)^a^	CC_50_ (μM)^b^
**1**–**10**	> 200	> 1500
**11**	112.7	2196.8
**12**	26.9	1565.5
**13**	106.7	1623.9
**14**	28.8	1451.7
Ribavirin	112.9	1517.5

The inhibition effects on influenza virus A/PR8/34 (H1N1) (MOI = 1.0) multiplication in MDCK cells were evaluated by virus yield reduction assay

^a^Inhibition concentration 50% (IC_50_): concentration required to inhibit influenza virus A/PR8/34 (H1N1) yield at 48 h post-infection by 50%. Values are mean ± SD (n = 4)

^b^Cytotoxic concentration 50% (CC_50_): concentration required to reduce MDCK cell viability by 50%. Values are mean ± SD (n = 3)

### Site-directed mutagenesis of VioA

The broad substrate promiscuity of VioA makes itself an excellent candidate for further enzyme engineering to generate diverse VLPs. We next set out to further investigate the biosynthetic potentials of VioA in vitro. However, no soluble VioA was obtained after exploring different conditions (data not shown). Therefore, we turned to probe its function by expressing different versions of site-mutated *vioA* in *S. coelicolor* M1146. Firstly, we did multiple sequence alignment of VioA with selected reported type III PKSs (Additional file 1: Figure S16). With the purpose to probe the substrate promiscuity and/or to improve catalytic efficiency of VioA, I174 and L190 (corresponding to T197 and I214 in VvSTS, respectively), as well as Y229 and S242 (corresponding to H261 and M274 in Gcs, respectively) were substituted with Ala, Ile, Ala and Ala, respectively. Structure modeling was simultaneously performed as described in the Materials and Methods section to help understanding the underlying mechanism. As shown in Additional file 1: Figure S17A, both I174A (ii) and Y229A (iv) displayed severe defection on VLPs production, supporting their important roles in substrate binding (I174) and cyclization (Y229), respectively (Additional file 1: Figure S17B). No change was observed for L190I (Additional file 1: Figure S17A, iii), indicating this substitution probably had no influence on the cyclization pocket (Additional file 1: Figure S17B). Conversely, VLPs production was completely abolished in S242A (Additional file 1: Figure S17A, v), demonstrating S242 to be essential for the activity of VioA, which was consistent with its position being close to the cyclization pocket in the structural model (Additional file 1: Figure S17B).

### P450 inhibitor increased the yields of anti-H1N1 VLPs by blocking side-chain oxidation

**Fig. 5:**
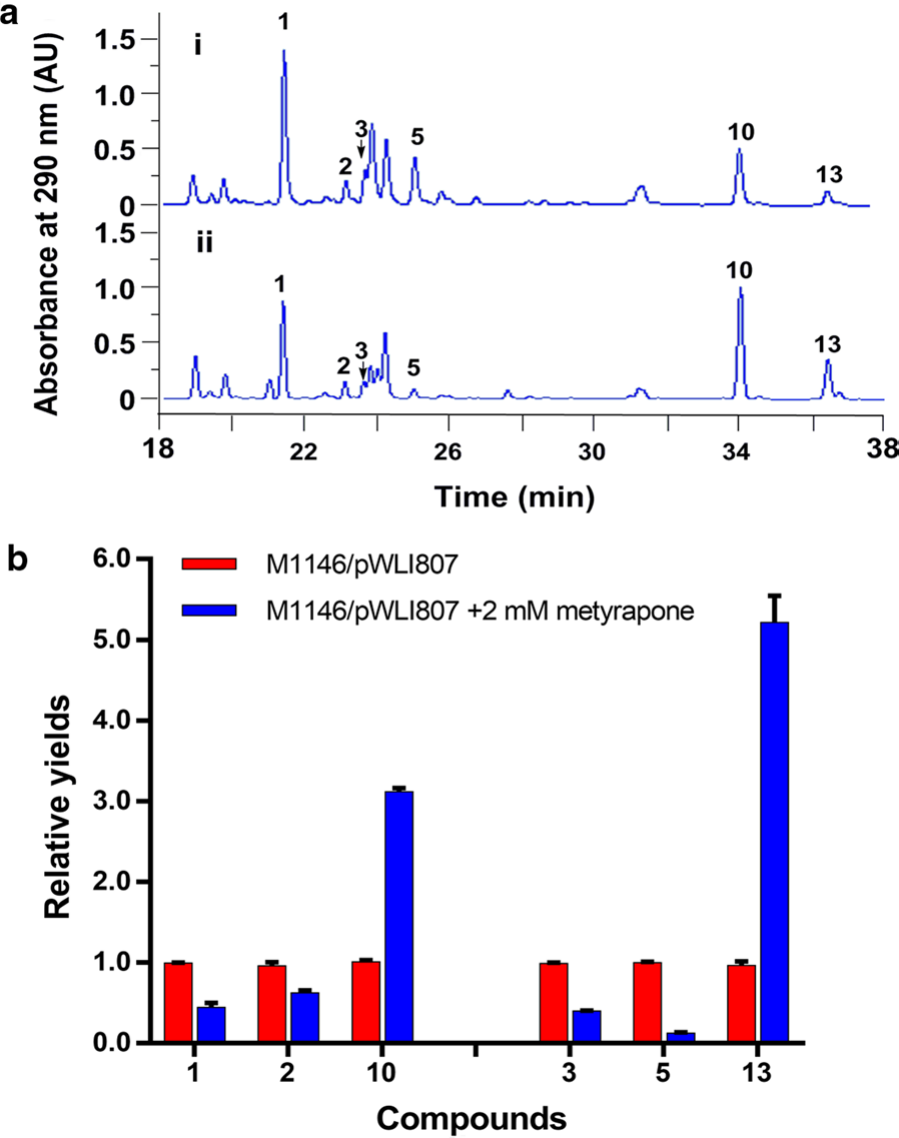
**a** HPLC traces of the fermentation broths from M1146/pWLI807 without metyrapone (i) and M1146/pWLI807 addition of 2 mM metyrapone (ii). **b** Relative yields of compounds **1**–**3**, **5**, **10** and **13** from M1146/pWLI807 without metyrapone and M1146/pWLI807 addition of 2 mM metyrapone

**Fig. 6:**
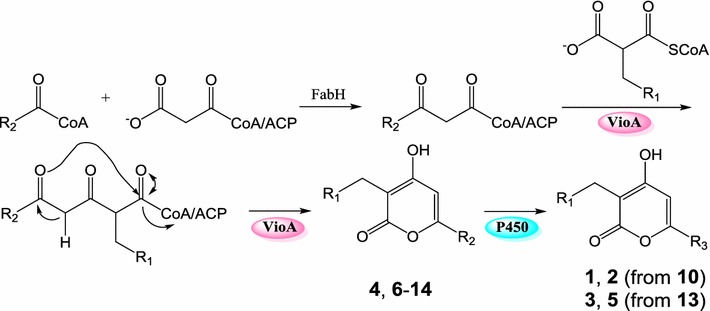
Proposed biosynthetic pathway of compounds **1**–**14**

The above bioassay results indicated that the presence of hydroxyl- (**1**–**3**) or keto-group (**5**) in the alkyl side chain at C-6 has negative impact on the bioactivity. The introduction of hydroxyl- or keto-group might happen before (as an oxidized starter unit) or after the assembly of the pyrone ring (as a tailoring step). To test if they are assembled by cytochrome P450 monooxygenases, 2 mM of P450 inhibitor metyrapone was added into the fermentation medium. The results (Fig. 5) showed that the production of compounds **1**–**3** and **5** were decreased by ~ 2.3-, ~ 1.4-, ~ 3-, and ~ 7.8-fold, respectively, and simultaneously, the yields of **10** and **13** were increased by ~twofold and ~fourfold, respectively. Conversely, no obvious changes of the other compounds were observed. This result indicated that the presence of hydroxyl- or keto-group is assembled by an unknown P450 located in the genome of the heterologous host as proposed in Fig. 6.

## Discussion

Heterologous expression serves as a proven effective approach for activating silent secondary metabolites gene clusters [28–31]. As type III PKSs are simple homodimer ketosynthases, they are especially convenient to be manipulated. In this study, the type III PKS gene *vioA* from deepsea-derived *S. somaliensis* SCSIO ZH66 was put under the control of the constitutive promoter P_gapDH_ followed by introduction into three different expression hosts. The accumulation of VLP products with different profiles in these three hosts (Fig. 3) supported the importance of precursor availability as well as genetic backgrounds of expression hosts.

The structural diversity of the VLPs compounds accumulated in *S. coelicolor* M1146/pWLI807 indicated that VioA can condense CoA- or ACP-tethered β-keto acids of different chain length with both ethylmalonyl CoA and methylmalonyl CoA, similar to that of Gcs [32]. However, compared to the data reported so far, VioA might recognize more diverse CoA- or ACP-tethered β-keto acids from fatty acid metabolism than Gcs. In the present study, although we tried to broaden the substrate promiscuity and/or improve catalytic efficiency of VioA via mutagenesis based on sequence alignment as well as previously mutagenesis results [14, 17], it is not surprising that no variants with expected properties were obtained. The VvSTS variants T197A and L214I were able to produce polyketides with different profiles than those of the wild-type VvSTS by changing the sizes of the substrate binding pocket and the cyclization pocket [14]; however, the corresponding substitutions in VioA led to severely impaired activity (I174A, Additional file 1: Figure S17A, ii) and no impact at all (L190I, Additional file 1: Figure S17A, iii). In Gcs, replacements of H261 and M274 with Ala significantly increased the yields of triketide pyrones [17]; on the contrary, the corresponding variants Y229A and S242A of VioA displayed severely impaired (Additional file 1: Figure S17A, iv) and totally abolished activity (Additional file 1: Figure S17A, v), respectively. The structure model of VioA supported the important roles of these mutated residues (Additional file 1: Figure S17B). Crystallographic studies would be contribute to disclose the underlying molecular basis for the substrate promiscuity of VioA and provide reliable guidance for further optimization.

Herein, for the first time, VLPs were shown to display anti-H1N1 virus activity (Table 3). Except for compound **12** harboring a 3-ethyl-4-hydroxy-α-pyrone ring, compounds **1**–**11**, **13** and **14** all have a 3-methyl-4-hydroxy-α-pyrone backbone but with diverse side chains at C-6. The differences in their activity can be ascribed to the influence of the substituent at C-3 and the alkyl side chain at C-6. The anti-H1N1 activity increased with decrease in the polarity of the compounds, suggesting that the lipophilic nature of the alkyl chain plays an important role for the activity, which is consistent with their anti-MRSA assay results [18]. These findings indicated prospective directions for improving anti-H1N1 activity of VLPs.

## Conclusion

The expression of *vioA* in *S. coelicolor* M1146 led to production of fourteen VLP compounds (**1**–**14**), among which five (**2**–**4**, **7** and **12**) are novel compounds and four (**11**–**14**) display anti-H1N1 activities. Here, for the first time, VLPs were shown to display antiviral activity. The production of VLPs derivatives with enhanced antiviral activity were increased by adding P450 inhibitor-metyrapone. The site-directed mutagenesis results of VioA would provide reference for future enzyme engineering.

## Methods

### Strains and plasmids

All strains and plasmids used in this study are listed in Additional file 1: Table S1. *Escherichia coli* DH5α was used as the host for general subcloning [33]. *E. coli* ET12567/pUZ8002 [34] was used as the cosmid donor host for *E. coli*–*Streptomyces* intergeneric conjugation. The deepsea-derived *S. somaliensis* SCSIO ZH66 has been described previously [18, 24]. *S. coelicolor* M1146 [22] and *S. sanyensis* FMA [23] were used as the host strains for heterologous expression. Plasmid extractions and DNA purifications were carried out using standardized commercial kits (OMEGA, Bio-Tek, Guangzhou, China). PCR reactions were carried out with primers listed in Additional file 1: Table S2 using Pfu DNA polymerase (TIANGEN, Beijing, China). Oligonucleotide synthesis and DNA sequencing were performed by Sunny Biotech company (Shanghai, China). Restriction endonucleases and T4 DNA ligase were purchased from Fermentas (Shenzhen, China).

### Growth conditions

*Escherichia coli* strains were routinely cultured in Luria–Bertani (LB) liquid medium at 37 °C, 200 rpm, or LB agar plate at 37 °C, with appropriate antibiotic selection. *Streptomyces* strains were grown at 30 °C on MS medium (3% soya flour, 2% mannitol, 2% agar powder) for sporulation and conjugation, and were cultured in Tryptic Soy Broth (TSB) medium (3% tryptic soya both, 10.3% sucrose, 0.1% tryptone, 0.05% yeast extract) for genomic DNA preparation. Fermentation medium consists of 1% soluble starch, 2% glucose, 4% corn syrup, 1% yeast extract, 0.3% beef extract, 0.05% MgSO_4_·7H_2_O, 0.05% KH_2_PO_4_, 0.2% CaCO_3_, and 3% sea salt, pH = 7.0.

### Bioinformatic analysis

The evolutionary history was inferred using the Neighbor-Joining method [35]. The optimal tree with the sum of branch length = 10.17229024 is shown. The percentage of replicate trees in which the associated taxa clustered together in the bootstrap test (1000 replicates) are shown next to the branches. The tree is drawn to scale, with branch lengths in the same units as those of the evolutionary distances used to infer the phylogenetic tree. The evolutionary distances were computed using the Poisson correction method and are in the units of the number of amino acid substitutions per site. 29 protein sequences were used for analysis. All positions containing gaps and missing data were eliminated. There were a total of 301 positions in the final dataset. Evolutionary analyses were conducted in MEGA5 [36].

### Overexpression of VioA

The *vioA* gene and the constitutive promoter P_gapDH_ were amplified from the genome of *S. somaliensis* SCSIO ZH66 using primer pairs of *vioA*FP/*vioA*RP*Bam*HI and P_gapDH_FP*Eco*RI/P_gapDH_RP (Additional file 1: Table S2), respectively. After digestion with *Bam*HI and *Eco*RI, the PCR products were cloned into the same sites of pMT3 and were confirmed by sequencing. The resulting plasmid pWLI902 was passed through *E. coli* ET12567/pUZ8002 and was then introduced into *S. coelicolor* M1146, *S. sanyensis* FMA and *S. somaliensis* SCSIO ZH66, respectively, via conjugation, according to the established procedures. Apramycin-resistant exconjugants were selected to afford *S. coelicolor* M1146/pWLI902, *S. sanyensis* FMA/pWLI902 and *S. somaliensis* SCSIO ZH66/pWLI902, respectively.

### Production and analyses of VLPs

Spores of *Streptomyces* strains were inoculated into 50 mL of medium in a 250 mL flask for production analysis or into 200 mL in a 1 L flask for isolation, and were incubated at 30 °C, 220 r.p.m for 7 days. The culture supernatants were extracted twice with an equal volume of EtOAc. The combined EtOAc extracts were concentrated *in vacuo* to afford a brown residue, which was dissolved in MeOH, filtered through a 0.2 μm filter, and subjected to HPLC analysis. The HPLC system consisted of Agilent 1260 Infinity Quaternary pumps and a 1260 Infinity diode-array detector. Analytical HPLC was performed on an Eclipse C18 column (5 μm, 4.6 × 150 mm) developed with a linear gradient from 5% to 80% B/A in 40 min (phase A: 0.1% HCOOH in H_2_O; phase B: 100% ACN supplemented with 0.1% HCOOH) followed by an additional 10 min at 100% B at flow rate of 1 mL/min and UV detection at 290 nm. A total volume of 23 L fermentation cultures were harvested by centrifugation. The supernatant was treated as above and 3.95 g of brown residue was obtained, which was applied to reversed-phase C18 open column, eluting with a gradient eluent of H_2_O–MeOH (from 9:1 to 1:19 and finally 100% MeOH) to collect 17 fractions. Fraction 3 (366.21 mg) was further subjected to semipreparative HPLC using a YMC ODS-A column (250 × 20 mm i.d, 5 μm) by a linear gradient from 60 to 75% B/A in 80 min (phase A: 0.1% HCOOH in H_2_O; phase B: 100% MeOH supplemented with 0.1% HCOOH) to afford compound **1** (15.02 mg), compound **2** (5.74 mg), compound **3** (5.72 mg), compound **4** (1.70 mg) and compound **5** (5.05 mg). Compound **6** (3.97 mg) was obtained by further separation of fraction 5 (90.00 mg) eluting with 65% MeOH in H_2_O supplemented with 0.1% HCOOH. Compound **7** (3.20 mg), compound **8** (8.36 mg) and compound **9** (3.36 mg) were obtained by further separation of fraction **7** (53.37 mg) eluting with 75% MeOH in H_2_O supplemented with 0.1% HCOOH. Compound **10** (37.05 mg), compound **11** (3.88 mg) and compound **13** (2.58 mg) were purified from fraction 8 (152.11 mg) eluting with 80% MeOH in H_2_O supplemented with 0.1% HCOOH. Compound **12** (2.00 mg) and compound **14** (2.30 mg) were purified from fraction 9 (36.15 mg) by a linear gradient from 70% to 100% B/A in 60 min (phase A: 0.1% HCOOH in H_2_O; phase B: 100% MeOH supplemented with 0.1% HCOOH). The identities of VLPs were elucidated by HR-ESI-MS and NMR analysis. HR-ESI-MS was carried out on Thermo LTQ-XL mass spectrometer. NMR data was recorded with an Agilent-DD2500 spectrometer.

### Site-directed mutation

For this work, the site-directed mutagenesis was created by overlapping primer mutagenesis [37]. To make each mutation, pairs of overlapping oligonucleotides, Additional file 1: Table S2, were synthesized. The first round of PCR was done using each of two mutagenic oligonucleotides and each of two (flanking) oligonucleotides complementary either to the 5′ or 3′ ends of the P_gapDH_::*vioA*. The two resulting PCR products were mixed, annealed and extended by few PCR cycles. The resulting Gel-purified full-length PCR products were cloned into pMT3 and confirmed by DNA sequencing.

### Protein structure modeling

The structural model of VioA was done by using I-TASSER server (http://zhanglab.ccmb.med.umich.edu/I-TASSER) [38]. The *C*-score for the VioA model is 1.15, indicating a high degree of structural homology to the templates, which is additionally confirmed by the low RMSD of 4.1 ± 2.8 Å. COACH was then used for protein–ligand-binding site prediction [39, 40].

### Biological assays

The cytotoxicity of compounds was measured by the MTT (3-[4,5-dimethylthiazol-2-yl]-2,5-diphenyl tetrazolium bromide; Sigma–Aldrich, USA) assay. Confluent MDCK cell cultures in 96-well plates were exposed to different concentrations of compounds in triplicate, using incubation conditions equivalent to those used in the antiviral assays. Next, 10 μL of PBS containing MTT (final concentration: 0.5 mg/mL) was added to each well. After 4 h incubation at 37 °C, the supernatant was removed and 200 μL of DMSO was added to each well to solubilize the formazan crystals. After vigorous shaking, absorbance values were measured in a microplate reader (Bio-Rad, USA) at 570 nm. The CC_50_ was calculated as the compound concentration necessary to reduce cell viability by 50%. Compounds **1**–**14** were evaluated for their anti-influenza A (H1N1) virus activities by the cytopathic effect (CPE) inhibition assay [26]. Madin–Darby canine kidney (MDCK) cells were obtained from Cell Bank of Chinese Academy of Sciences (Shanghai, China) and grown in RPM1640 medium supplemented with 10% FBS, 100 units/mL of penicillin and 100 μg/mL of streptomycin. Influenza virus (A/Puerto Rico/8/34 [H1N1]; PR/8) was propagated in 10-day-old embryonated eggs for 3 days at 36.5 °C. MDCK cell cultures in 96-well plates were firstly infected with H1N1 virus (MOI = 0.1), and were then treated with different compounds in triplicate after removal of the virus inoculum. After 48 h incubation at 37 °C, the cells were fixed with 4% formaldehyde for 20 min at room temperature. After removal of the formaldehyde, the cells were stained with 0.1% crystal violet for 30 min. The plates were washed and dried, and the intensity of crystal violet staining for each well was measured at 570 nm. The virus inhibition (%) was calculated by the equation:

$${\text{Virus inhibition }}\left( \% \right) \, = \, \left[ {\left( {{\text{A}}_{{{\text{sample 57}}0}} - {\text{ A}}_{{{\text{virus 57}}0}} } \right) \, / \, \left( {{\text{A}}_{{{\text{mock 57}}0 \, - }} {\text{A}}_{{{\text{virus 57}}0}} } \right)} \right] \times 100;$$

where A_mock 570_ was the absorbance without virus infection, A_sample 570_ was absorbance with virus infection and drug treatment, A_virus 570_ was absorbance with virus infection but without drugs. Ribavirin injection (50 mg/mL) as a positive control was obtained from LuKang Cisen (China).

## Additional file

**Additional file 1: Table S1.** Plasmids and strains used in this study. **Table S2.** Primer pairs used in this study. **Table S3.** Homologous locus of *vioAB* in different *Streptomyces* genomes. **Figure S1.** Relative yields for compounds **1**–**14** in different strains. **Figure S2.** Spectral data of **1**. **Figure S3**. Spectral data of **2**. **Figure S4.** Spectral data of **3**. **Figure S5.** Spectral data of **4**. **Figure S6.** Spectral data of **5**. **Figure S7.** Spectral data of **6**. **Figure S8.** Spectral data of **7**. **Figure S9.** Spectral data of **8**. **Figure S10.** Spectral data of **9**. **Figure S11.** Spectral data of **10**. **Figure S12.** Spectral data of **11**. **Figure S13.** Spectral data of **12**. **Figure S14.** Spectral data of **13**. **Figure S15.** Spectral data of **14**. **Figure S16.** Multiple-sequence alignments of VioA with selected type III PKSs. **Figure S17.** Site-directed mutagenesis study of VioA.

## Abbreviations

PKS: polyketide synthase
VLP: violapyrone
CHS: chalcone synthase
OKS: octaketide synthase
VvSTS: *Vitis vinifera* stilbene synthase
Sg-RppA: *Streptomyces griseus* RppA
Sc-RppA: *Streptomyces coelicolor* RppA
Gcs: germicidin synthase
*wbl*: *whiB*-like
MRSA: methicillin-resistant *Staphylococcus aureus*
Cpz6: caprazamycin 6
DpyA: dihydroxyphenylglycine A
ACP: acyl carrier protein
NMR: nuclear magnetic resonance
HPLC: high-pressure liquid chromatography
HR-ESI-MS: high-resolution electrospray mass spectrometry
COSY: correlation spectroscopy
HSQC: heteronuclear singular quantum correlation
HMBC: heteronuclear multiple bond correlation
CPE: cytopathic effect
IC_50_: half maximal inhibitory concentration
UV: ultra violet
MS: mannitol-soy flour
LB: Luria–Bertani
TSB: tryptic soy broth
MDCK: Madin–Darby canine kidney
