# Supplementary material for: Overexpression of a type III PKS gene affording novel violapyrones with enhanced anti-influenza A virus activity
Source: Microb Cell Fact. 2018 Apr 12;17:61. doi: 10.1186/s12934-018-0908-9 (PMC5898002; doi:10.1186/s12934-018-0908-9)
Supplement: Supplementary file 1 — Additional file 1: Table S1. Plasmids and strains used in this study. Table S2. Primer pairs used in this study. Table S3. Homologous locus of vioAB in different Streptomyces genomes. Figure S1. Relative yields for compounds 1–14 in different strains. Figure S2. Spectral data of 1. Figure S3. Spectral data of 2. Figure S4. Spectral data of 3. Figure S5. Spectral data of 4. Figure S6. Spectral data of 5. Figure S7. Spectral data of 6. Figure S8. Spectral data of 7. Figure S9. Spectral data of 8. Figure S10. Spectral data of 9. Figure S11. Spectral data of 10. Figure S12. Spectral data of 11. Figure S13. Spectral data of 12. Figure S14. Spectral data of 13. Figure S15. Spectral data of 14. Figure S16. Multiple-sequence alignments of VioA with selected type III PKSs. Figure S17. Site-directed mutagenesis study of VioA. [file 12934_2018_908_MOESM1_ESM.docx]

**Overexpression of a type III PKS gene affording novel violapyrones with enhanced anti-influenza A virus activity**

Lukuan Hou^1^, Huiming Huang^1^, Huayue Li^1,2^, Shuyao Wang^1^, Jianhua Ju^3^ and Wenli Li^1,2^*

^1^ Key Laboratory of Marine Drugs, Ministry of Education of China, School of Medicine and Pharmacy, Ocean University of China, Qingdao 266003, China

^2^ Laboratory for Marine Drugs and Bioproducts of Qingdao National Laboratory for Marine Science and Technology, Qingdao 266237, China

^3^ CAS Key Laboratory of Tropical Marine Bioresources and Ecology, Guangdong Key Laboratory of Marine Materia Medica, RNAM Center for Marine Microbiology, South China Sea Institute of Oceanology, Chinese Academy of Sciences, 164 West Xingang Road, Guangzhou 510301, China

***** Author to whom correspondence should be addressed

Email addresses:

LH: houlukuan1991@163.com

HH: hmhuang1988@163.com

HL: lihuayue@ouc.edu.cn

SW: [shuyaowang224@126.com](mailto:shuyaowang224@126.com)

JJ: jju@scsio.ac.cn

WL: [liwenli@ouc.edu.cn](mailto:liwenli@ouc.edu.cn)

**Table of contents**

| No. |  | Page |
| --- | --- | --- |
| 1． | **Table S1.** Plasmids and strains used in this study. | S3 |
| 2. | **Table S2.** Primer pairs used in this study. | S3 |
| 3. | **Table S3.** Homologous *locus* of *vioAB* in different *Streptomyces* genomes. | S4 |
| 4. | **Figure S1.** Relative yields for compounds **1**-**14** in different strains. | S5 |
| 5. | **Figure S2**. Spectral data of **1**. | S6 |
| 6. | **Figure S3.** Spectral data of **2**. | S7 |
| 7. | **Figure S4.** Spectral data of **3**. | S10 |
| 8. | **Figure S5.** Spectral data of **4**. | S13 |
| 9. | **Figure S6.** Spectral data of **5**. | S16 |
| 10. | **Figure S7.** Spectral data of **6**. | S17 |
| 11. | **Figure S8.** Spectral data of **7**. | S18 |
| 12. | **Figure S9.** Spectral data of **8**. | S21 |
| 13. | **Figure S10.** Spectral data of **9**. | S22 |
| 14. | **Figure S11.** Spectral data of **10**. | S23 |
| 15. | **Figure S12.** Spectral data of **11**. | S24 |
| 16. | **Figure S13.** Spectral data of **12**. | S25 |
| 17 | **Figure S14.** Spectral data of **13**. | S28 |
| 18 | **Figure S15.** Spectral data of **14**. | S29 |
| 19 | **Figure S16.** Multiple-sequence alignments of VioA with selected type III PKSs. | S30 |
| 20 | **Figure S17.** Site-directed mutagenesis study of VioA. | S31 |
| 21 | **References** | S32 |

**Table S1**. Plasmids and strains used in this study.

| Plasmids or strains | Description | Reference or source |
| --- | --- | --- |
| Plasmids |  |  |
| pWLI801 | cosmid harboring *vio* genes from *S. somaliensis* SCSIO ZH66 | [1] |
| pWLI806 | pMT3, Integrative vector, derivative of pSET152 | [2] |
| pWLI807 | pWLI806 carrying *vioA* gene from pWLI801, under the control of P_gapDH_ | This study |
| pWLI808 | pWLI807 derivative where I174 of *vioA* gene was mutanted to alanine | This study |
| pWLI809 | pWLI807 derivative where L190 of *vioA* gene was mutanted to isoleucine | This study |
| pWLI810 | pWLI807 derivative where Y229 of *vioA* gene was mutanted to alanine | This study |
| pWLI811 | pWLI807 derivative where S242 of *vioA* gene was mutanted to alanine | This study |
| Strains |  |  |
| *E. coli* DH5*a* | Host strain for general cloning | Stratagene |
| *E. coli* ET12567/pUZ8002 | Host strain for conjugation | [3] |
| *S. somaliensis* SCSIO ZH66 | Wild type, isolated from deepsea sendiment | [4] |
| *S. coelicolor* M1146 | Host strain for overexpression | [5] |
| *S. sanyensis* FMA | Host strain for overexpression | [6] |
| ZH66/pWL806 | *S. somaliensis* SCSIO ZH66 with pWL806 | This study |
| M1146/ pWL806 | *S. coelicolor* M1146 with pWL806 | This study |
| FMA/ pWL806 | *S. sanyensis* FMA with pWL806 | This study |
| ZH66/pWLI807 | *S. somaliensis* SCSIO ZH66 with pWL807 | This study |
| M1146/ pWLI807 | *S. coelicolor* M1146 with pWL807 | This study |
| FMA/ pWLI807 | *S. sanyensis* FMA with pWL807 | This study |
| M1146/ pWLI808 | *S. coelicolor* M1146 with pWL808 | This study |
| M1146/ pWLI809 | *S. coelicolor* M1146 with pWL809 | This study |
| M1146/ pWLI810 | *S. coelicolor* M1146 with pWL810 | This study |
| M1146/ pWLI811 | *S. coelicolor* M1146 with pWL811 | This study |

**Table S2**. Primer pairs used in this study.

| Primer name | Sequence (5'-3') |
| --- | --- |
| P_gapDH_ *Eco*RI F | CCGGAATTCcgcaccccctggtcaacgcg |
| P_gapDH_ R | gagccgatctcctcgttggt |
| *vioA* F | atggccatccacatcgccca |
| *vioABam*HI R | CGCGGATCCtcacgccgcccagaccccac |
| I174A F | aggtcatctccgcggcatacaaccacgccga |
| I174A R | tcggcgtggttgtatgccgcggagatgacct |
| L190I F | tgatctacaaggcgattttcggggacagtgc |
| L190I R | gcactgtccccgaaaatcgccttgtagatca |
| Y229A F | acagcctcacccgggcagtcggccgcatcga |
| Y229A R | tcgatgcggccgactgcccgggtgaggctgt |
| S242A F | gcctccacttcgacgcaacgaaggaagccct |
| S242A R | agggcttccttcgttgcgtcgaagtggaggc |

**Table S3**. Homologous *locus* of *vioAB* in different *Streptomyces* genomes.

|  |  |  | ***vioA*** | | | |  | ***vioB*** | | |
| --- | --- | --- | --- | --- | --- | --- | --- | --- | --- | --- |
| **No.** | **strains** | **Description** | | | **Identity** | **Accession** | **Description** | | **Identity** | **Accession** |
| 1 | *Streptomyces somaliensis* | polyketide synthase | | 100% | | [AMN09000.1](https://www.ncbi.nlm.nih.gov/protein/1002342306?report=genbank&log$=prottop&blast_rank=1&RID=U7088GZF014) | transcriptional regulator | | 100% | [AMN09001.1](https://www.ncbi.nlm.nih.gov/protein/1002342307?report=genbank&log$=prottop&blast_rank=1&RID=U70DXRSW016) |
| 2 | *Streptomyces* sp. BvitLS-983 | PhlD | | 94% | | [WP_093561382.1](https://www.ncbi.nlm.nih.gov/protein/1225553697?report=genbank&log$=prottop&blast_rank=5&RID=U7088GZF014) | XRE family transcriptional regulator | | 94% | [WP_093561384.1](https://www.ncbi.nlm.nih.gov/protein/1225553699?report=genbank&log$=prottop&blast_rank=3&RID=U70DXRSW016) |
| 3 | *Streptomyces* sp. ZL12 | polyketide synthase | | 74% | | [WP_012821850.1](https://www.ncbi.nlm.nih.gov/protein/502581100?report=genbank&log$=prottop&blast_rank=13&RID=U7088GZF014) | XRE family transcriptional regulator | | 64% | [WP_012821849.1](https://www.ncbi.nlm.nih.gov/protein/502581099?report=genbank&log$=prottop&blast_rank=23&RID=U70DXRSW016) |
| 4 | *Streptomyces griseus* | polyketide synthase | | 96% | | [WP_033239937.1](https://www.ncbi.nlm.nih.gov/protein/702687281?report=genbank&log$=prottop&blast_rank=2&RID=U7088GZF014) | transcriptional regulator | | 97% | [WP_078944382.1](https://www.ncbi.nlm.nih.gov/protein/1155039343?report=genbank&log$=prottop&blast_rank=5&RID=U70DXRSW016) |
| 5 | *Streptomyces* sp. 150FB | polyketide synthase | | 64% | | [WP_040026276.1](https://www.ncbi.nlm.nih.gov/protein/748774685?report=genbank&log$=prottop&blast_rank=35&RID=U7088GZF014) | XRE family transcriptional regulator | | 68% | [WP_040026277.1](https://www.ncbi.nlm.nih.gov/protein/748774686?report=genbank&log$=prottop&blast_rank=6&RID=U70DXRSW016) |
| 6 | *Streptomyces glaucescens* | PhlD | | 72% | | [WP_086732872.1](https://www.ncbi.nlm.nih.gov/protein/1197794422?report=genbank&log$=prottop&blast_rank=14&RID=U7088GZF014) | XRE family transcriptional regulator | | 68% | [WP_086732871.1](https://www.ncbi.nlm.nih.gov/protein/1197794421?report=genbank&log$=prottop&blast_rank=7&RID=U70DXRSW016) |
| 7 | *Streptomyces griseoluteus* | polyketide synthase | | 66% | | [WP_030214297.1](https://www.ncbi.nlm.nih.gov/protein/663175682?report=genbank&log$=prottop&blast_rank=16&RID=U7088GZF014) | XRE family transcriptional regulator | | 61% | [WP_030214298.1](https://www.ncbi.nlm.nih.gov/protein/663175683?report=genbank&log$=prottop&blast_rank=39&RID=U70DXRSW016) |
| 8 | *Streptomyces* sp. FXJ1.172 | PhlD | | 64% | | [WP_067038692.1](https://www.ncbi.nlm.nih.gov/protein/1055375471?report=genbank&log$=prottop&blast_rank=26&RID=U7088GZF014) | XRE family transcriptional regulator | | 65% | [WP_067038694.1](https://www.ncbi.nlm.nih.gov/protein/1055375473?report=genbank&log$=prottop&blast_rank=9&RID=U70DXRSW016) |
| 9 | *Streptomyces* sp. NRRL S-920 | polyketide synthase | | 63% | | [WP_030777516.1](https://www.ncbi.nlm.nih.gov/protein/664245599?report=genbank&log$=prottop&blast_rank=43&RID=U7088GZF014) | XRE family transcriptional regulator | | 66% | [WP_030777519.1](https://www.ncbi.nlm.nih.gov/protein/664245602?report=genbank&log$=prottop&blast_rank=10&RID=U70DXRSW016) |
| 10 | *Streptomyces leeuwenhoekii* | polyketide synthase | | 64% | | [WP_047121282.1](https://www.ncbi.nlm.nih.gov/protein/824055856?report=genbank&log$=prottop&blast_rank=20&RID=U7088GZF014) | XRE family transcriptional regulator | | 68% | [WP_047121283.1](https://www.ncbi.nlm.nih.gov/protein/824055857?report=genbank&log$=prottop&blast_rank=11&RID=U70DXRSW016) |
| 11 | *Streptomyces* sp. MUSC 14 | PhlD | | 61% | | [WP_071372930.1](https://www.ncbi.nlm.nih.gov/protein/1100232489?report=genbank&log$=prottop&blast_rank=40&RID=U7088GZF014) | XRE family transcriptional regulator | | 64% | [WP_071372931.1](https://www.ncbi.nlm.nih.gov/protein/1100232490?report=genbank&log$=prottop&blast_rank=12&RID=U70DXRSW016) |
| 12 | *Streptomyces* | polyketide synthase | | 61% | | [WP_030362413.1](https://www.ncbi.nlm.nih.gov/protein/663363970?report=genbank&log$=prottop&blast_rank=45&RID=U7088GZF014) | XRE family transcriptional regulator | | 64% | [WP_030362414.1](https://www.ncbi.nlm.nih.gov/protein/663363971?report=genbank&log$=prottop&blast_rank=15&RID=U70DXRSW016) |
| 13 | *Streptomyces* | polyketide synthase | | 75% | | [WP_031030004.1](https://www.ncbi.nlm.nih.gov/protein/664512271?report=genbank&log$=prottop&blast_rank=11&RID=U7088GZF014) | XRE family transcriptional regulator | | 65% | [WP_031030001.1](https://www.ncbi.nlm.nih.gov/protein/664512268?report=genbank&log$=prottop&blast_rank=16&RID=U70DXRSW016) |
| 14 | *Streptomyces* sp. MUSC 1 | PhlD | | 62% | | [WP_071384146.1](https://www.ncbi.nlm.nih.gov/protein/1100243880?report=genbank&log$=prottop&blast_rank=36&RID=U7088GZF014) | XRE family transcriptional regulator | | 64% | [WP_071384145.1](https://www.ncbi.nlm.nih.gov/protein/1100243879?report=genbank&log$=prottop&blast_rank=17&RID=U70DXRSW016) |
| 15 | *Streptomyces* | polyketide synthase | | 64% | | [WP_030658478.1](https://www.ncbi.nlm.nih.gov/protein/664121408?report=genbank&log$=prottop&blast_rank=25&RID=U7088GZF014) | XRE family transcriptional regulator | | 64% | [WP_030658475.1](https://www.ncbi.nlm.nih.gov/protein/664121405?report=genbank&log$=prottop&blast_rank=20&RID=U70DXRSW016) |
| 16 | *Streptomyces silvensis* | PhlD | | 59% | | [WP_058848919.1](https://www.ncbi.nlm.nih.gov/protein/970980188?report=genbank&log$=prottop&blast_rank=46&RID=U7088GZF014) | XRE family transcriptional regulator | | 63% | [WP_058848780.1](https://www.ncbi.nlm.nih.gov/protein/970980049?report=genbank&log$=prottop&blast_rank=22&RID=U70DXRSW016) |
| 17 | *Streptomyces reticuli* | PhlD | | 64% | | [WP_059255760.1](https://www.ncbi.nlm.nih.gov/protein/974671495?report=genbank&log$=prottop&blast_rank=39&RID=U7088GZF014) | XRE family transcriptional regulator | | 64% | [WP_059255762.1](https://www.ncbi.nlm.nih.gov/protein/974671497?report=genbank&log$=prottop&blast_rank=26&RID=U70DXRSW016) |
| 18 | *Streptomyces* sp. HmicA12 | polyketide synthase | | 60% | | [WP_026245475.1](https://www.ncbi.nlm.nih.gov/protein/648553724?report=genbank&log$=prottop&blast_rank=47&RID=U7088GZF014) | XRE family transcriptional regulator | | 64% | [WP_018528903.1](https://www.ncbi.nlm.nih.gov/protein/517353411?report=genbank&log$=prottop&blast_rank=29&RID=U70DXRSW016) |
| 19 | *Streptomyces kanamyceticus* | PhlD | | 66% | | [WP_055546752.1](https://www.ncbi.nlm.nih.gov/protein/943911351?report=genbank&log$=prottop&blast_rank=17&RID=U7088GZF014) | XRE family transcriptional regulator | | 64% | [WP_079043447.1](https://www.ncbi.nlm.nih.gov/protein/1158511585?report=genbank&log$=prottop&blast_rank=30&RID=U70DXRSW016) |
| 20 | *Streptomyces* sp. ATexAB-D23 | hypothetical protein | | 72% | | [WP_018552903.1](https://www.ncbi.nlm.nih.gov/protein/517378551?report=genbank&log$=prottop&blast_rank=12&RID=U7088GZF014) | XRE family transcriptional regulator | | 63% | [WP_018552902.1](https://www.ncbi.nlm.nih.gov/protein/517378550?report=genbank&log$=prottop&blast_rank=32&RID=U70DXRSW016) |
| 21 | *Streptomyces* sp. CB02460 | PhlD | | 73% | | [WP_073967002.1](https://www.ncbi.nlm.nih.gov/protein/1121988162?report=genbank&log$=prottop&blast_rank=8&RID=U7088GZF014) | XRE family transcriptional regulator | | 62% | [WP_073967001.1](https://www.ncbi.nlm.nih.gov/protein/1121988161?report=genbank&log$=prottop&blast_rank=33&RID=U70DXRSW016) |
| 22 | *Streptomyces aurantiacus* | polyketide synthase | | 64% | | [WP_016638252.1](https://www.ncbi.nlm.nih.gov/protein/514915599?report=genbank&log$=prottop&blast_rank=33&RID=U7088GZF014) | XRE family transcriptional regulator | | 62% | [WP_016638253.1](https://www.ncbi.nlm.nih.gov/protein/514915600?report=genbank&log$=prottop&blast_rank=36&RID=U70DXRSW016) |


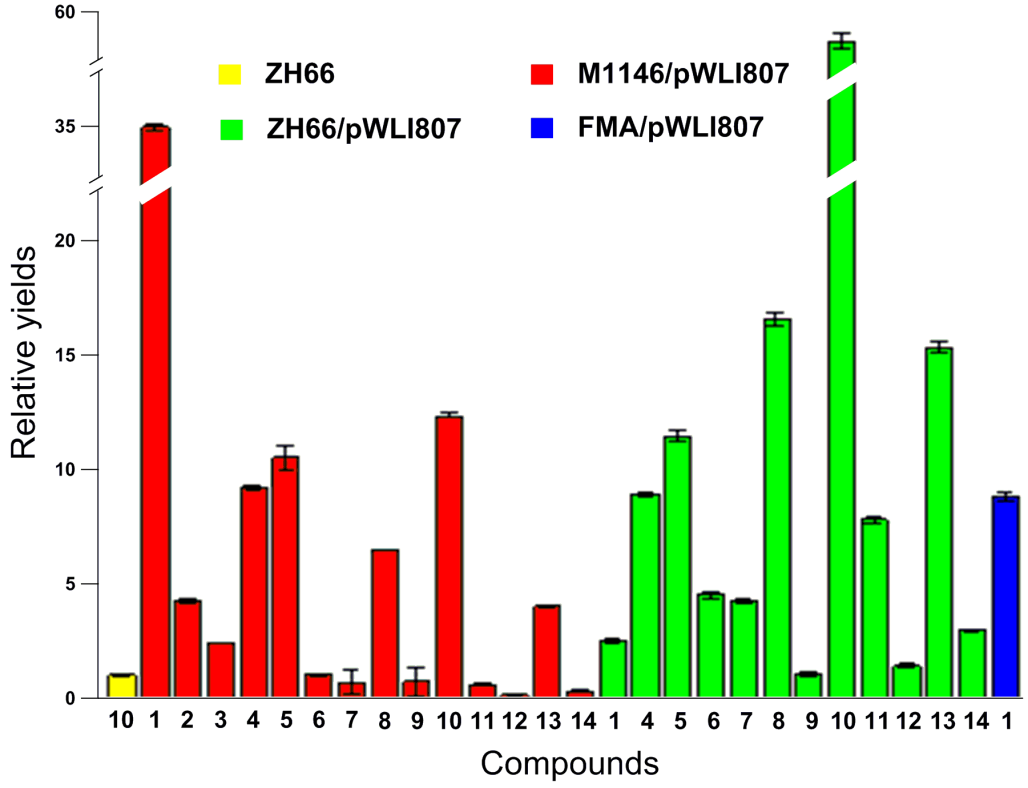


**Figure S1.** Relative yields for compounds **1**-**14** in different strains. Since all the compounds display almost identical UV spectrum, the relative yields were calculated based on peak area of each compound in comparison with that of compound **10** in the wild type *S. somaliensis* ZH66.

**Figure S2**. Spectral data of **1**.

**Figure S2 (A).** The HRMS spectrum of **1**.

**
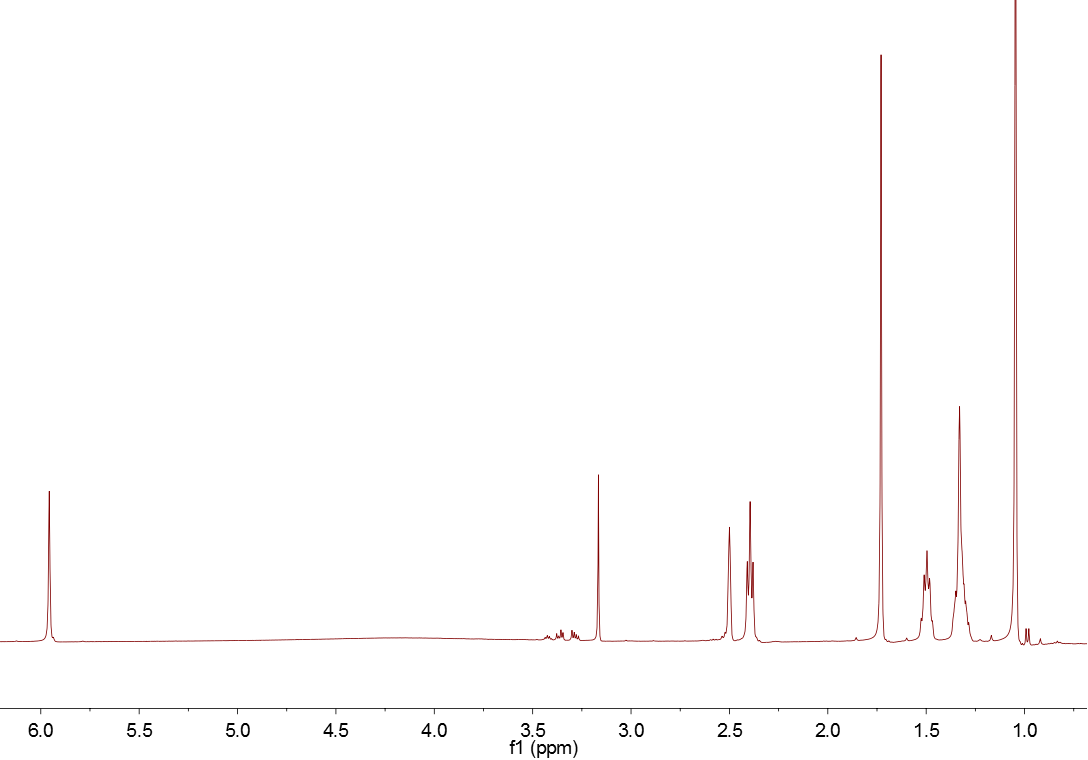
**

**Figure S2 (B).** ^1^H NMR (500 MHz, DMSO-*d_6_*) spectrum of **1**.

**Figure S3**. Spectral data of **2**.

**Figure S3 (A).** The HRMS spectrum of **2**.


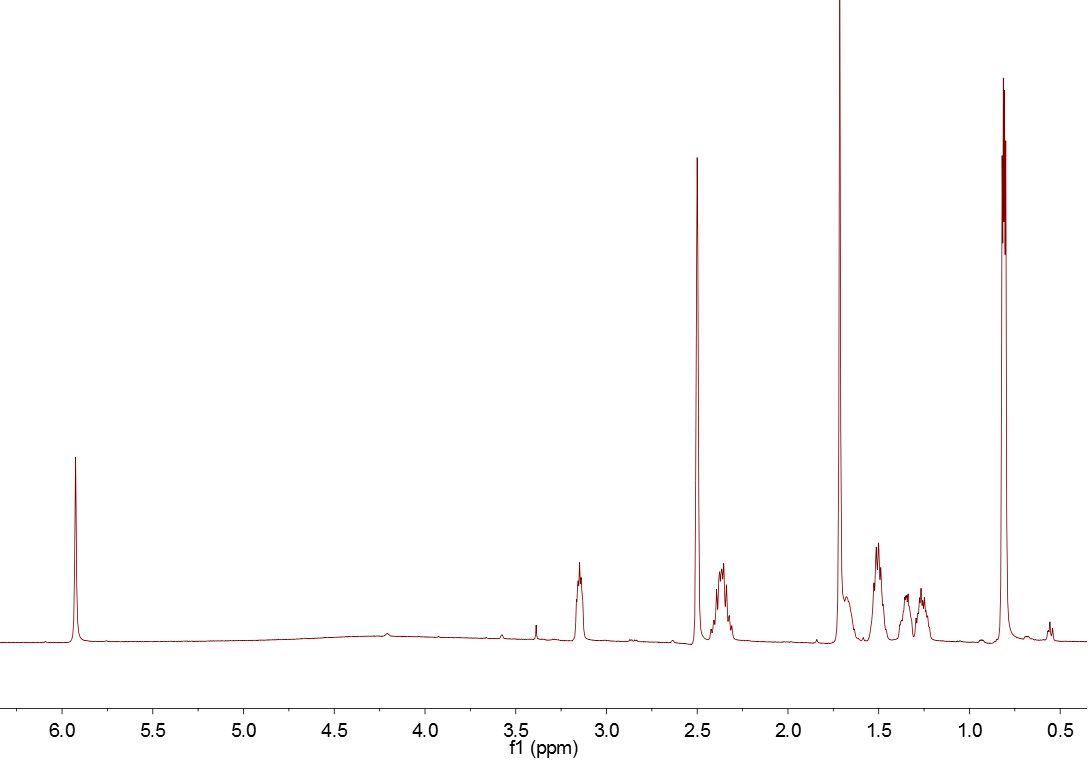


**Figure S3 (B).** ^1^H NMR (500 MHz, DMSO- *d_6_*) spectrum of **2**.


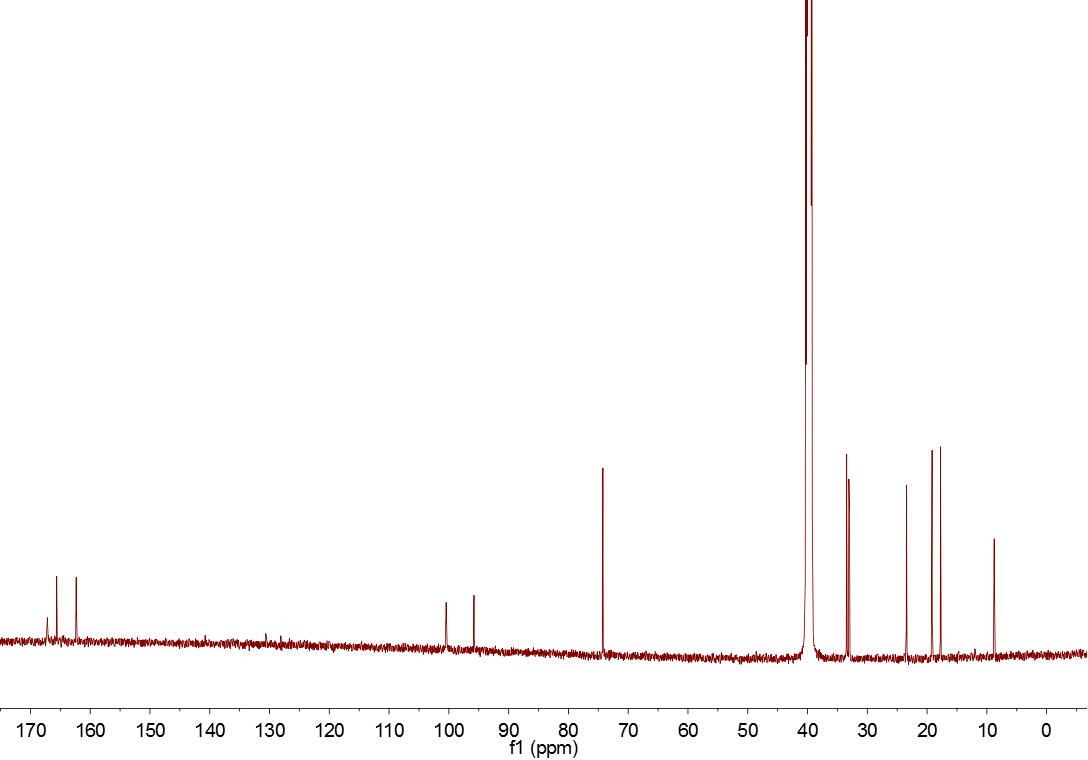


**Figure S3 (C).** ^13^C NMR (125 MHz, DMSO- *d_6_*) spectrum of **2**.

**Figure S3 (D).** The ^1^H-^1^H COSY spectrum of **2** in DMSO- *d_6_*.

**Figure S3 (E).** The HMBC spectrum of **2** in DMSO- *d_6_*.

**Figure S3 (F).** The HSQC spectrum of **2** in DMSO- *d_6_*.

**Figure S4**. Spectral data of **3**.

**Figure S4 (A).** The HRMS spectrum of **3**.

**Figure S4 (B).** ^1^H NMR (500 MHz, DMSO- *d_6_*) spectrum of **3**.

**Figure S4 (C).** The ^1^H-^1^H COSY spectrum of **3** in DMSO- *d_6_*.

**Figure S4 (D).** The HMBC spectrum of **3** in DMSO- *d_6_*.

**Figure S4 (E).** The HSQC spectrum of **3** in DMSO- *d_6_*.

**Figure S5**. Spectral data of **4**.

**Figure S5 (A).** The HRMS spectrum of **4**.


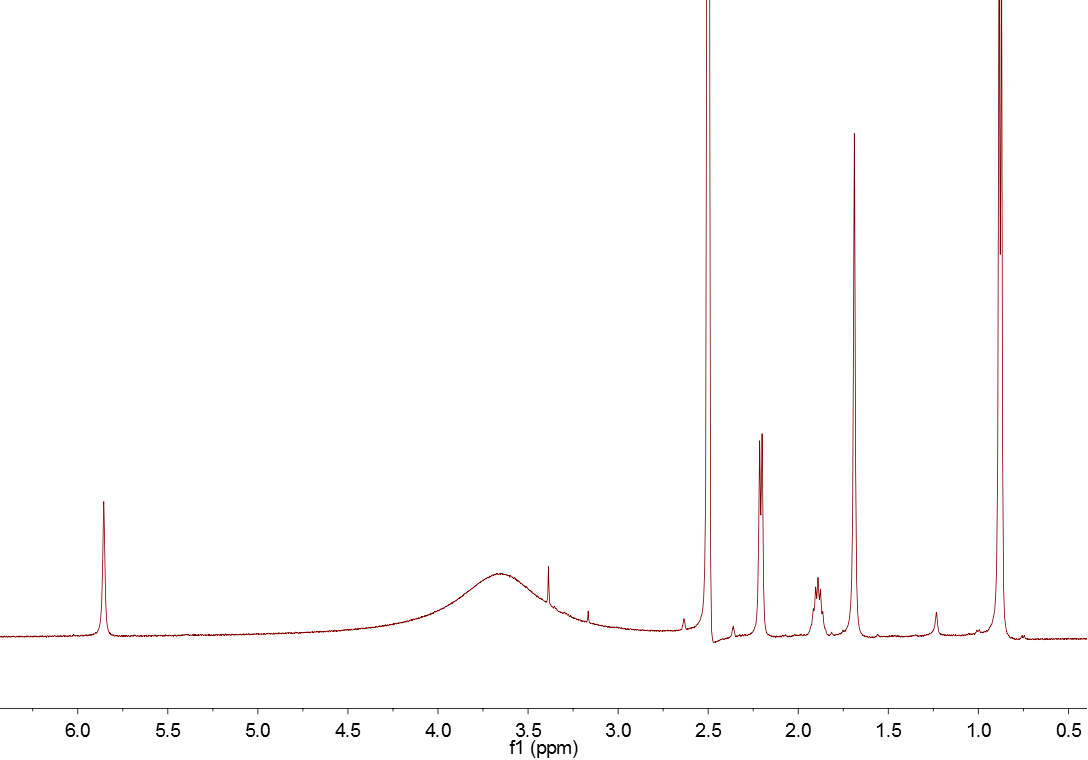


**Figure S5 (B).** ^1^H NMR (500 MHz, DMSO- *d_6_*) spectrum of **4**.


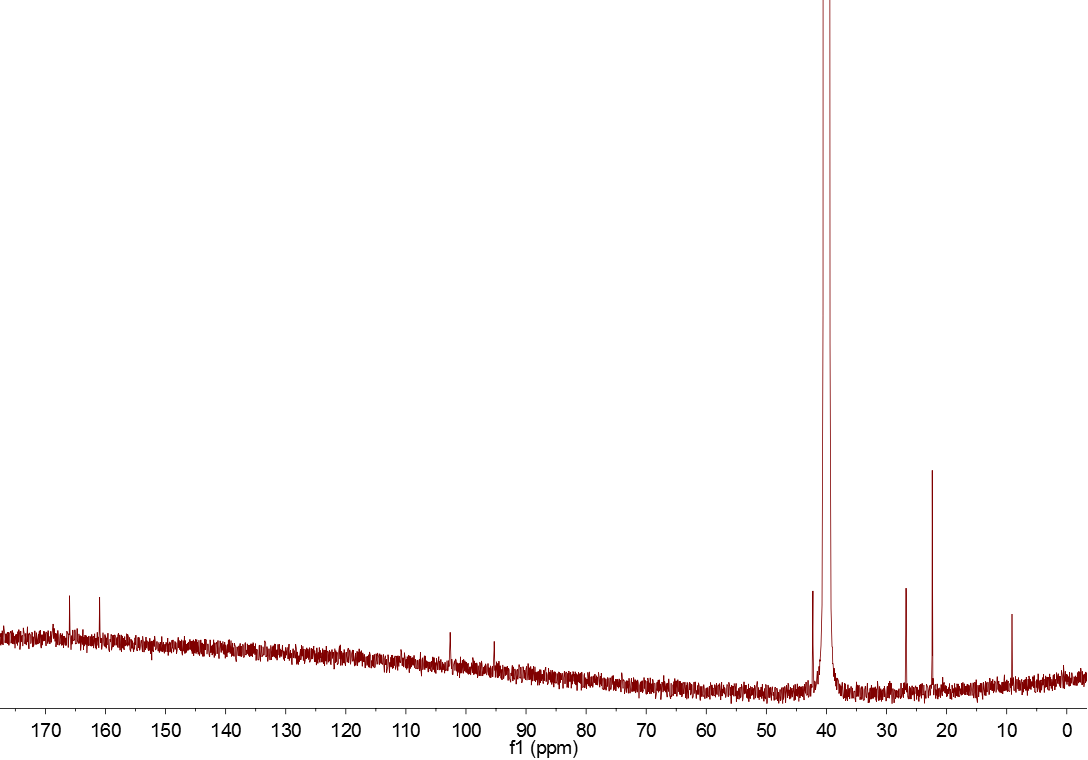


**Figure S5 (C).** ^13^C NMR (125 MHz, DMSO- *d_6_*) spectrum of **4**.

**Figure S5 (D).** The ^1^H-^1^H COSY spectrum of **4** in DMSO- *d_6_*.

**Figure S5 (E).** The HMBC spectrum of **4** in DMSO- *d_6_*.

**Figure S5 (F).** The HSQC spectrum of **4** in DMSO- *d_6_*.

**Figure S6**. Spectral data of **5**.

**Figure S6 (A).** The HRMS spectrum of **5**.


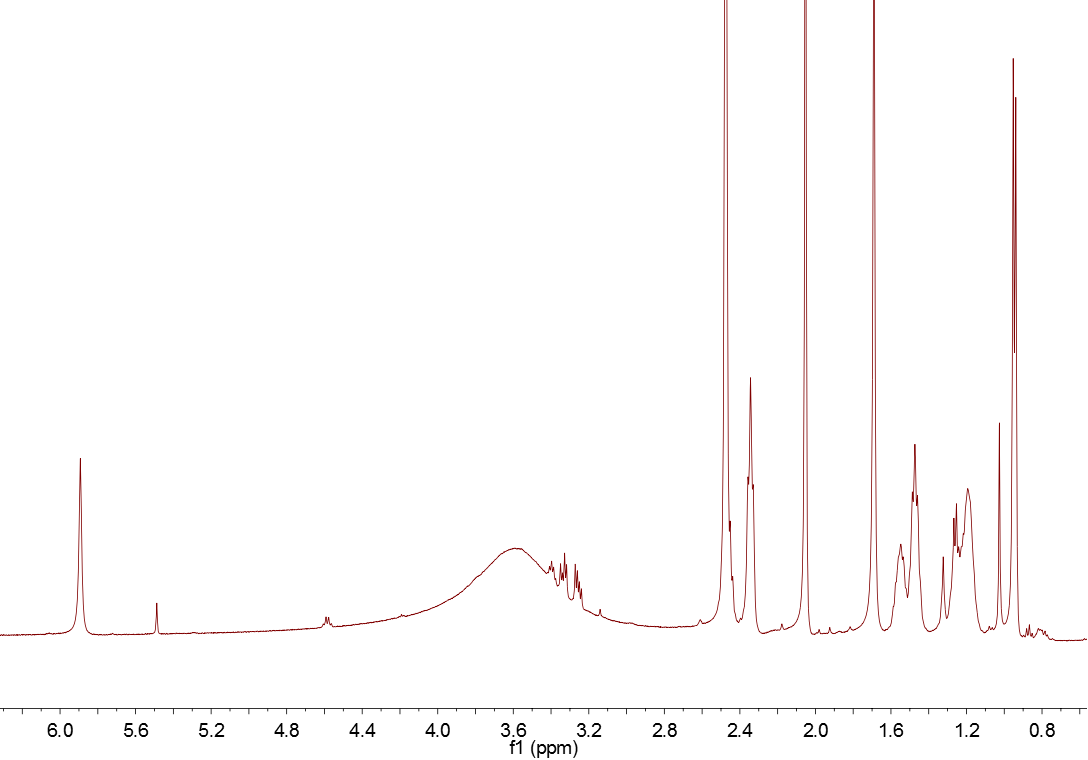


**Figure S6 (B).** ^1^H NMR (500 MHz, DMSO- *d_6_*) spectrum of **5**.

**Figure S7**. Spectral data of **6**.

**Figure S7 (A).** The HRMS spectrum of **6**.


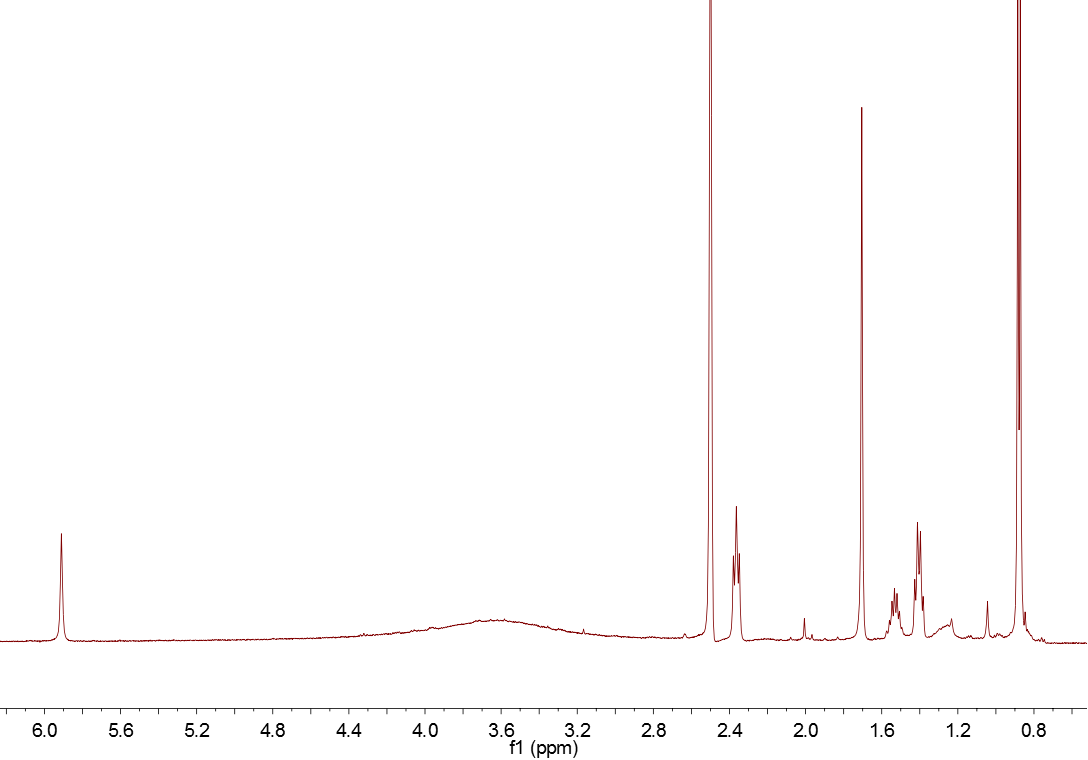


**Figure S7 (B).** ^1^H NMR (500 MHz, DMSO- *d_6_*) spectrum of **6**.

**Figure S8**. Spectral data of **7**.

**Figure S8 (A).** The HRMS spectrum of **7**.


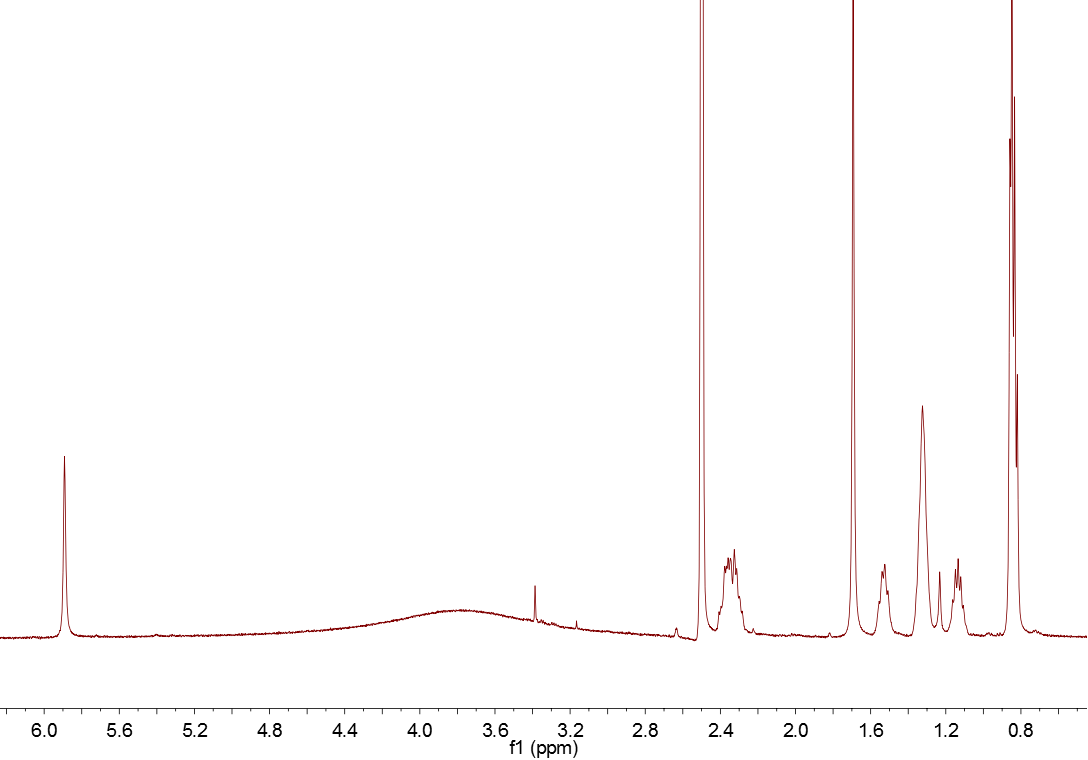


**Figure S8 (B).** ^1^H NMR (500 MHz, DMSO- *d_6_*) spectrum of **7**.


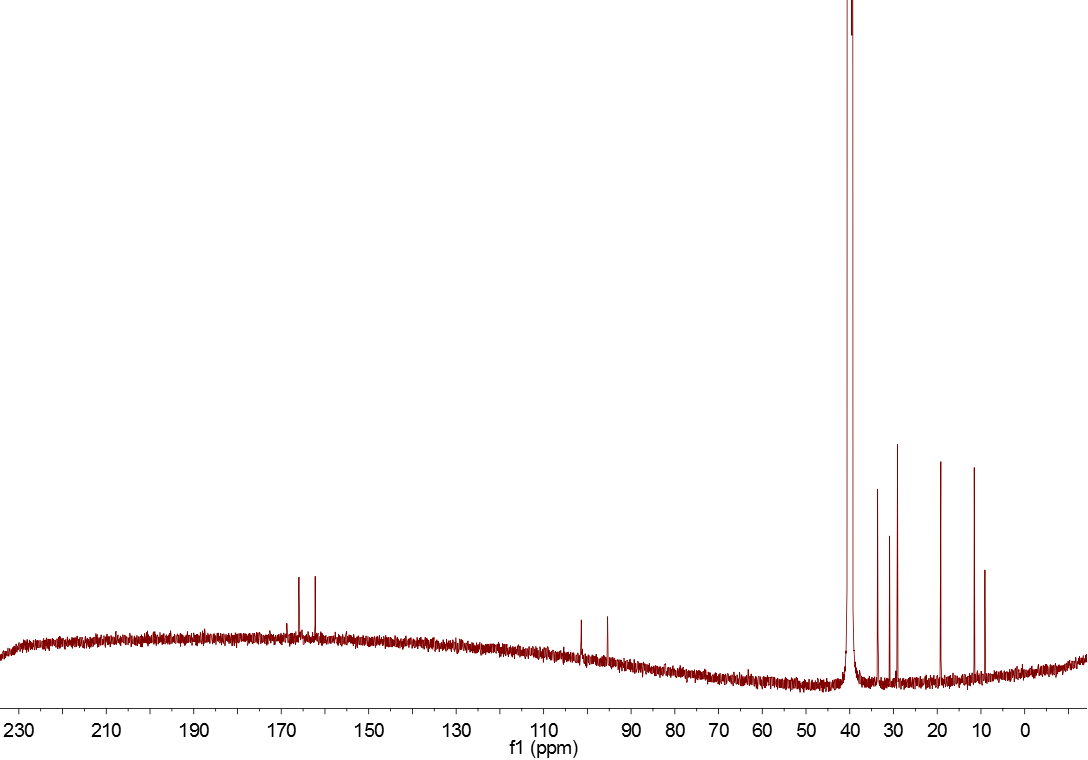


**Figure S8 (C).** ^13^C NMR (125 MHz, DMSO- *d_6_*) spectrum of **7**.

**Figure S8 (D).** The ^1^H-^1^H COSY spectrum of **7** in DMSO- *d_6_*.

**Figure S8 (E).** The HMBC spectrum of **7** in DMSO- *d_6_*.

**Figure S8 (F).** The HSQC spectrum of **7** in DMSO- *d_6_*.

**Figure S9**. Spectral data of **8**.

**Figure S9 (A).** The HRMS spectrum of **8**.

**Figure S9 (B).** ^1^H NMR (500 MHz, DMSO- *d_6_*) spectrum of **8**.

**Figure S10**. Spectral data of **9**.

**Figure S10 (A).** The HRMS spectrum of **9**.


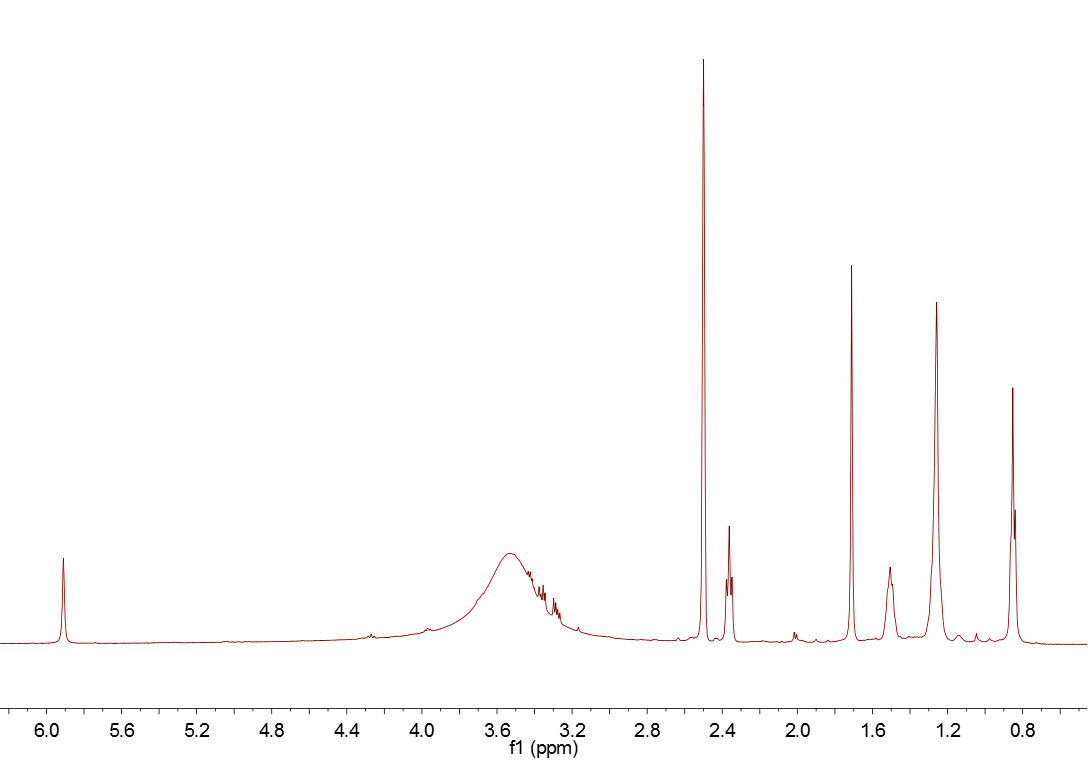
 **Figure S10 (B).** ^1^H NMR (500 MHz, DMSO- *d_6_*) spectrum of **9**.

**Figure S11**. Spectral data of **10**.

**Figure S11 (A).** The HRMS spectrum of **10**.


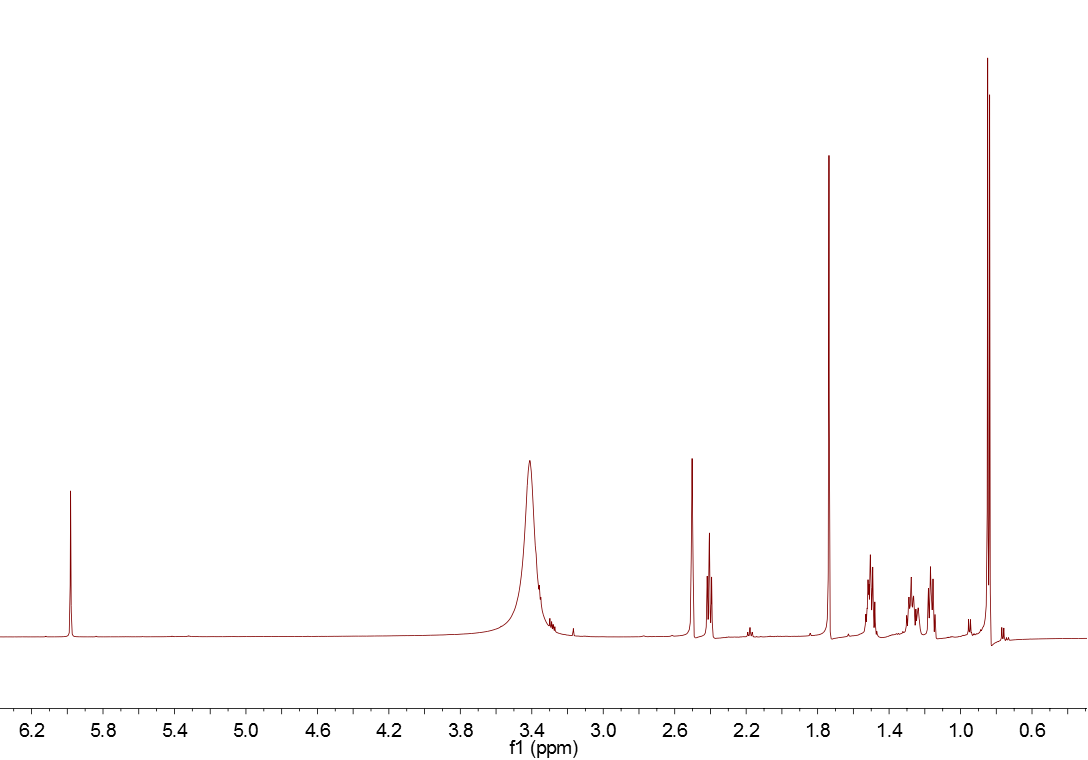


**Figure S11 (B).** ^1^H NMR (500 MHz, DMSO- *d_6_*) spectrum of **10**.

**Figure S12**. Spectral data of **11**.

**Figure S12 (A).** The HRMS spectrum of **11**.

**Figure S12 (B).** ^1^H NMR (500 MHz, DMSO- *d_6_*) spectrum of **11**.

**Figure S13**. Spectral data of **12**.

**Figure S13 (A).** The HRMS spectrum of **12**.

**
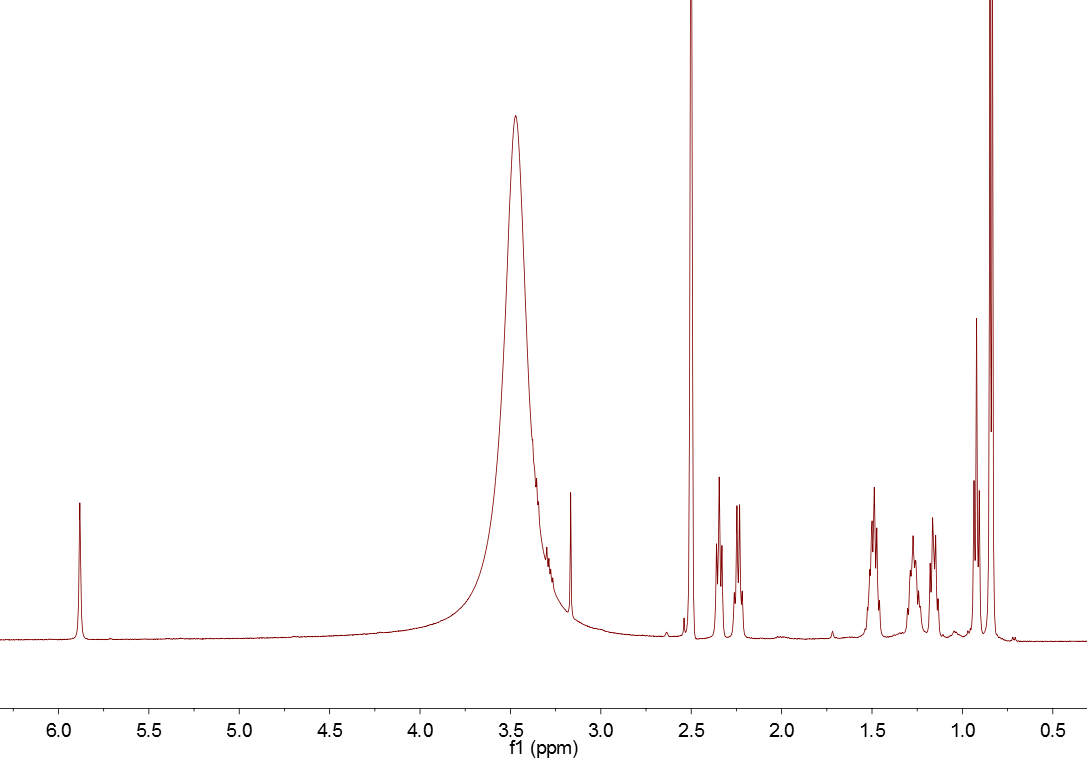
**

**Figure S13 (B).** ^1^H NMR (500 MHz, DMSO- *d_6_*) spectrum of **12**.

**
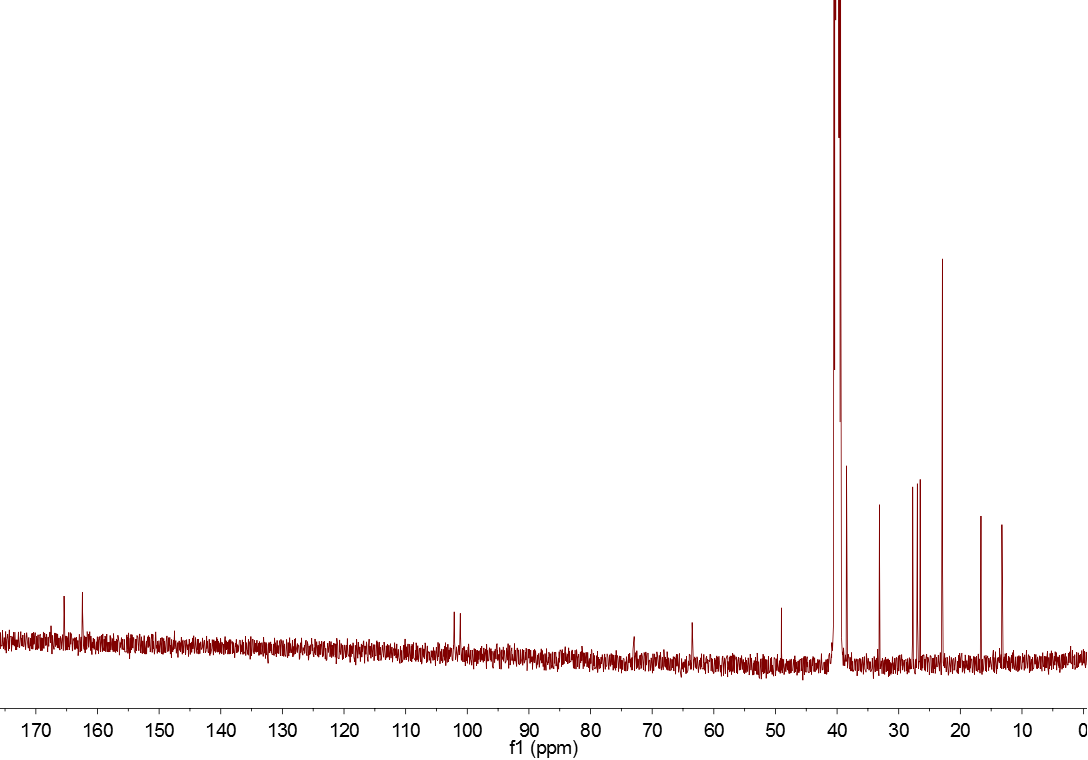
**

**Figure S13 (C).** ^13^C NMR (125 MHz, DMSO- *d_6_*) spectrum of **12**.

**Figure S13 (D).** The ^1^H-^1^H COSY spectrum of **12** in DMSO- *d_6_*.

**Figure S13 (E).** The HMBC spectrum of **12** in DMSO- *d_6_*.

**Figure S13 (F).** The HSQC spectrum of **12** in DMSO- *d_6_*.

**Figure S14**. Spectral data of **13**.

**Figure S14 (A).** The HRMS spectrum of **13**.


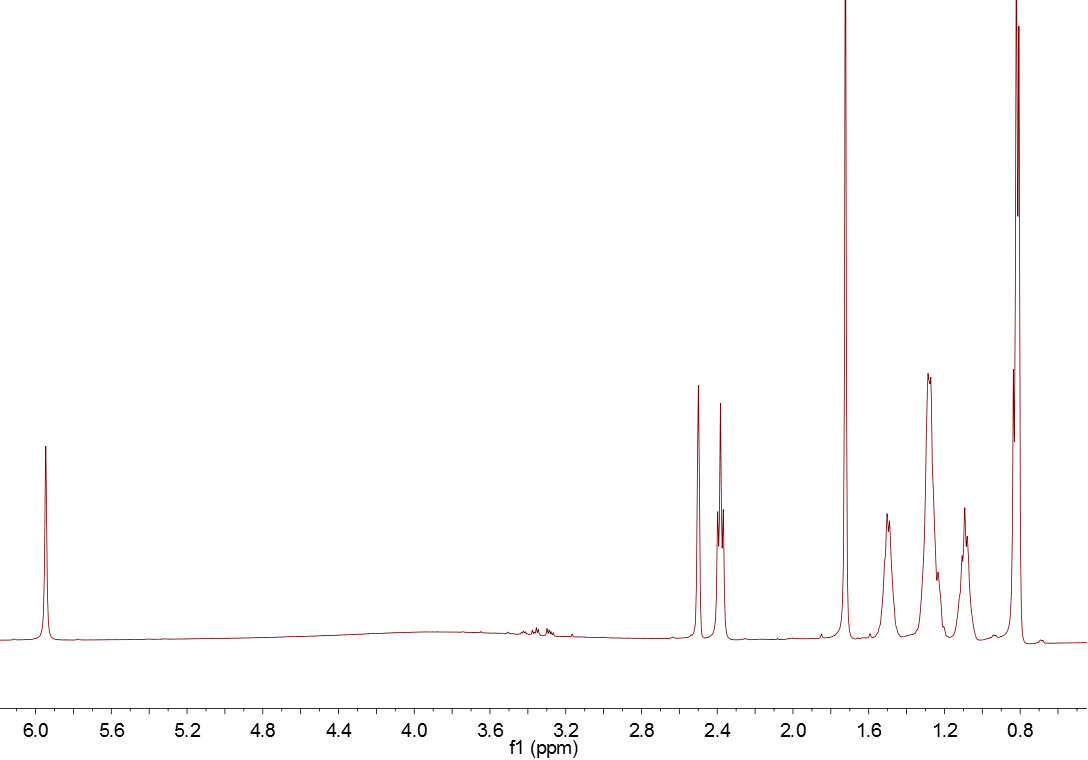


**Figure S14 (B).** ^1^H NMR (500 MHz, DMSO- *d_6_*) spectrum of **13**.

**Figure S15**. Spectral data of **14**.

**Figure S15 (A).** The HRMS spectrum of **14**.


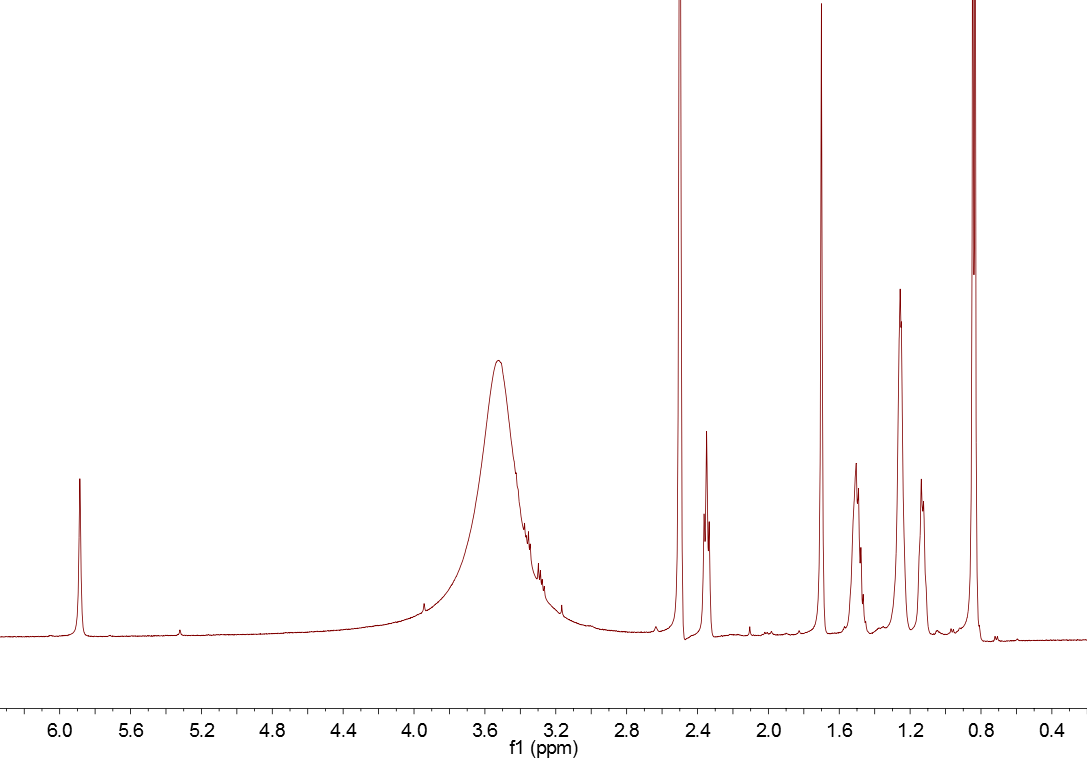
 **Figure S15 (B).** ^1^H NMR (500 MHz, DMSO- *d_6_*) spectrum of **14**.


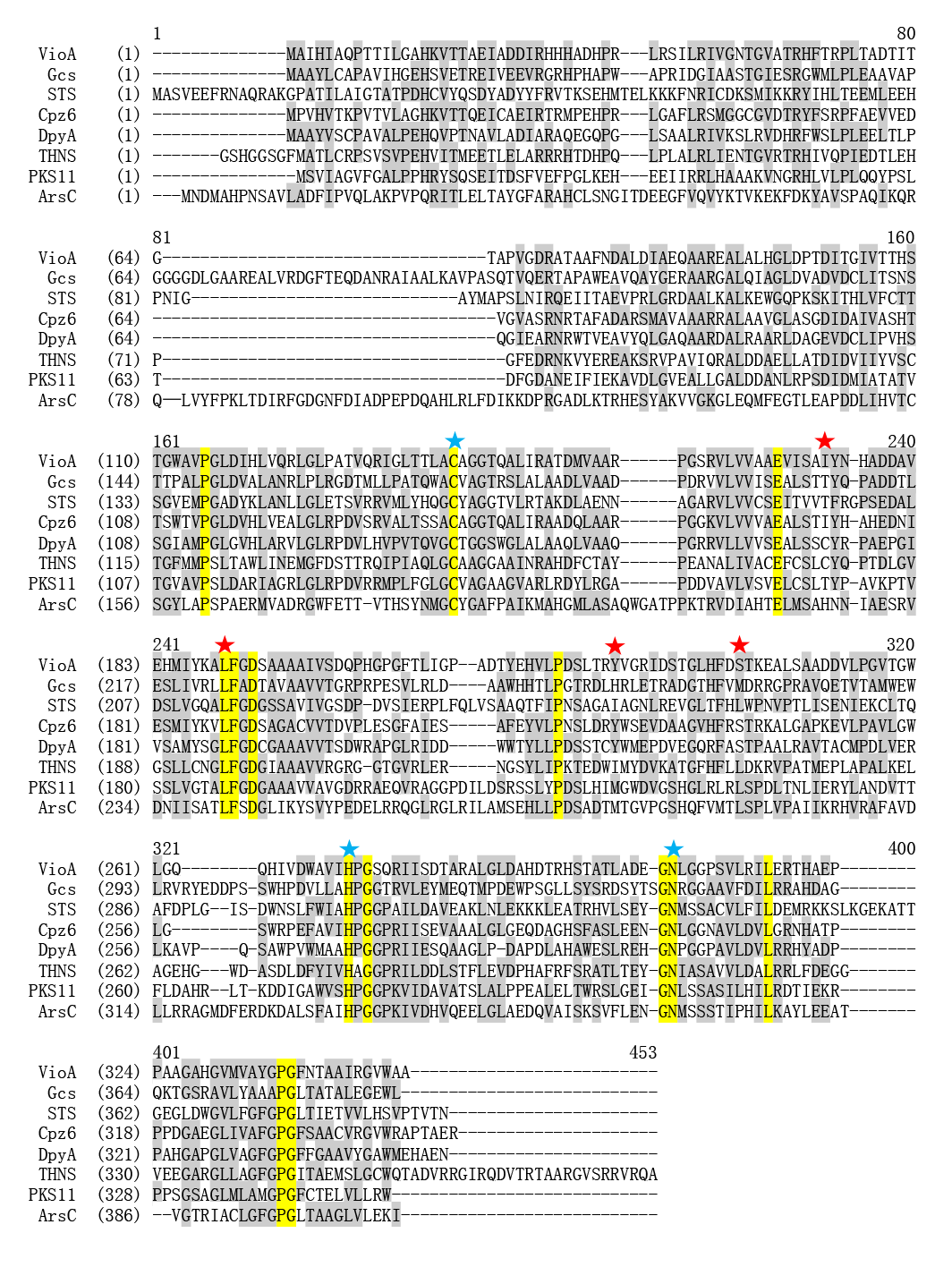


**Figure S16.** Multiple-sequence alignments of VioA with selected type III PKSs. The origins and accession numbers of the type III PKSs subjected to alignment are as follows: VioA, *S. somaliensis* (AMN09000); Gcs, *S. coelicor* (3V7I_A); STS, *Vitis vinifera* (BAB20980.1); Cpz6, *Streptomyces* sp. SN-1061M (ADC96652); DpyA, *Streptomyces* sp. SN-593 (BAQ19510); THNS, *S. coelicolor* (1U0M_A); PKS11, *Mycobacterium tuberculosis* (4JAO); ArsC, *Azotobacter vinelandii* (3VS8). Residues conserved among all aligned sequences are highlighted in a yellow background; residues subjected to mutagenesis are indicated with red stars. The Cys-His-Asn (CHN) catalytic triad is indicated with blue stars.

**(A) (B)**


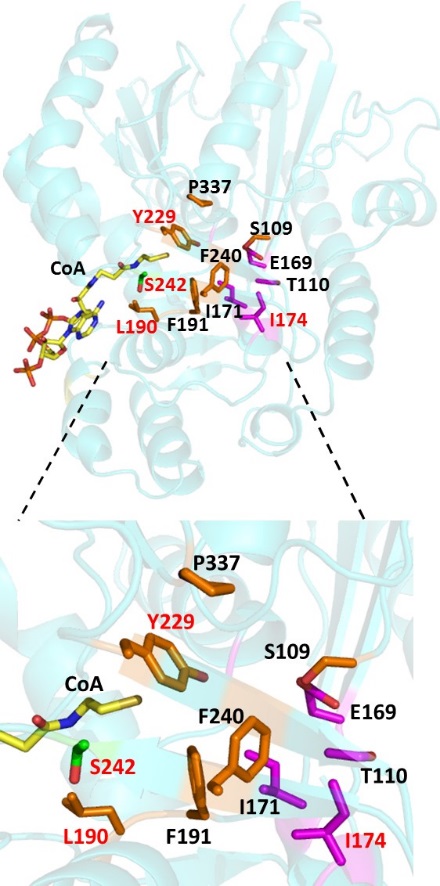


**Figure S17.** Site-directed mutagenesis study of VioA. (A) HPLC traces of the fermentation broths of *S. coelicolor* M1146 strains. (i) M1146/pWLI807 (WT); (ii) M1146/pWLI808 (I174A); (iii) M1146/pWLI809 (L190I); (iv) M1146/pWLI810 (Y229A); (v) M1146/pWLI811 (S242A). (B) Structure model of VioA. The residues (S109, L190, F191, Y229, F240 and P337) in the cyclization pocket are shown in brown; the residues (T110, E169, I171 and I174) in the substrate binding pocket are shown in purple [7]. Four residues subjected to mutagenesis (I174, L190, Y229, S242) are labelled in red. CoA was indicated in yellow.

**References**

1. Huang H, Hou L, Li H, Qiu Y, Ju J, Li W: Activation of a plasmid-situated type III PKS gene cluster by deletion of a *wbl* gene in deepsea-derived *Streptomyces somaliensis* SCSIO ZH66. *Microb Cell Fact* 2016, 15(1):116.

2. Bierman M, Logan R, O'Brien K, Seno ET, Nagaraja Rao R, Schoner BE: Plasmid cloning vectors for the conjugal transfer of DNA from *Escherichia coli* to *Streptomyces* spp. *Gene* 1992, 116(1):43-49.

3. Datsenko KA, Wanner BL: One-step inactivation of chromosomal genes in *Escherichia coli* K-12 using PCR products. *Proc Natl Acad Sci U S A* 2000, 97(12):6640-6645.

4. Zhang Y, Huang H, Xu S, Wang B, Ju J, Tan H, Li W: Activation and enhancement of Fredericamycin A production in deepsea-derived *Streptomyces somaliensis* SCSIO ZH66 by using ribosome engineering and response surface methodology. *Microb Cell Fact* 2015, 14:64.

5. Gomez-Escribano JP, Bibb MJ: Engineering *Streptomyces coelicolor* for heterologous expression of secondary metabolite gene clusters. *Microb Biotechnol* 2011, 4(2):207-215.

6. Li T, Du Y, Cui Q, Zhang J, Zhu W, Hong K, Li W: Cloning, characterization and heterologous expression of the indolocarbazole biosynthetic gene cluster from marine-derived *Streptomyces sanyensis* FMA. *Mar Drugs* 2013, 11(2):466-488.

7. Austin MB, Noel JP: The chalcone synthase superfamily of type III polyketide synthases. *Natural Product Reports* 2003, 20(1):79-110.
